# The immunobiology and therapeutic potential of regulatory T cells in autoimmune diseases and allergic diseases

**DOI:** 10.3389/fimmu.2025.1709915

**Published:** 2026-01-13

**Authors:** Wen-Wen Xie, Jian-Bin Huang, Yi-Chi Zhou, Jing-Yi Yuan, Jia-Xue Feng, Xiao-Hang Shi, Li Tian, Xian-Hai Zeng, Shu-Qi Qiu, Mei-Zhen Zhao, Bao-Hui Cheng, Hao-Tao Zeng

**Affiliations:** 1 Department of Graduate and Scientific Research, Zunyi Medical University, Zhuhai, Guangdong, China; 2 Department of Otolaryngology, Longgang Otolaryngology Hospital & Shenzhen Otolaryngology Research, Shenzhen, China; 3 Department of Gastroenterology, Beijing University of Chinese Medicine Shenzhen Hospital (Longgang), Shenzhen, China

**Keywords:** allergic diseases, autoimmune diseases, epigenetics, immunetolerance, regulatory T cells (Tregs), Treg plasticity

## Abstract

Autoimmune and allergic diseases represent two major categories of immune-mediated disorders that collectively impose a significant global health burden. Although driven by distinct triggers—aberrant responses against self-antigens in autoimmunity and hypersensitivity to innocuous environmental antigens in allergy—both classes of disease are fundamentally rooted in a failure of immunological tolerance. At the center of this regulatory failure lies the dysfunction of regulatory T cells (Tregs) which are the master orchestrators of peripheral tolerance, actively suppressing effector immune responses through the secretion of inhibitory cytokines and contact-dependent inhibition. In both autoimmune and allergic conditions, defects in Treg number, stability, or suppressive function permit the uncontrolled expansion of autoreactive lymphocytes in autoimmunity, while in allergic diseases, it fails to constrain the T helper 2 (Th2) cell-mediated pathways that drive pathology. Despite the well-established role of Tregs in each disease category, research often proceeds in parallel, leaving a critical knowledge gap regarding the convergent mechanisms of Treg failure across these interconnected pathologies. A unified understanding of how factors such as genetic predispositions and environmental influences cohesively impact Treg function remains underdeveloped. This review addresses this gap by providing a comprehensive synthesis of Treg immunobiology, with a specific emphasis on the convergent pathways that underpin their dysfunction in both autoimmune and allergic diseases. By elucidating the shared principles of Treg-mediated immune dysregulation, this review aims to provide a robust conceptual framework to accelerate the development of next-generation therapies capable of restoring tolerance across this broad spectrum of disorders.

## 1 Introduction

Autoimmune diseases and allergic diseases represent two major categories of immune-mediated disorders that collectively affect millions worldwide, imposing significant burdens on healthcare systems and quality of life (1). Autoimmune diseases, such as rheumatoid arthritis, multiple sclerosis, and type 1 diabetes, arise from aberrant immune responses targeting self-antigens, leading to chronic inflammation and tissue damage (2), allergic diseases, including asthma, atopic dermatitis, and food allergies, involve hypersensitivity reactions to innocuous environmental antigens, often mediated by type 2 immune pathways (3). Despite these apparent differences, both conditions share a common underlying mechanism: dysregulation of immune tolerance, where the immune system fails to appropriately suppress harmful responses (4, 5).

Central to this immune homeostasis are Tregs, a specialized subset of CD4^+^ T lymphocytes characterized by the expression of the transcription factor Foxp3, which orchestrate peripheral tolerance by suppressing effector T cell activation, cytokine production, and antigen-presenting cell function (6). Tregs exert their suppressive effects through multiple mechanisms, including the secretion of anti-inflammatory cytokines such as IL-10 and TGF-β, direct cell-cell contact via CTLA-4, and modulation of dendritic cell maturation. In healthy individuals, Tregs maintain a delicate balance, preventing autoimmunity by tolerizing self-reactive T cells and averting allergies by dampening responses to allergens. In autoimmune diseases, Treg dysfunction—manifested as reduced numbers, impaired suppressive capacity, or defective trafficking to inflamed tissues—contributes to the breakdown of self-tolerance, allowing autoreactive T and B cells to proliferate unchecked (7). Similarly, in allergic diseases, diminished Treg activity or altered Treg subsets fail to curb Th2-skewed responses, resulting in excessive IgE production, mast cell degranulation, and eosinophilic inflammation (8). A key commonality between these disorders lies in the shared role of Tregs in enforcing tolerance: both involve a failure of Treg-mediated suppression, albeit against distinct antigen types—self-antigens in autoimmunity and exogenous allergens in allergies (9). This overlap is further evidenced by genetic associations, such as polymorphisms in FoxP3 or IL-10 genes, which predispose individuals to both autoimmune and allergic pathologies, highlighting a unified immunoregulatory defect (10). Despite these parallels, research into the shared immunobiological roles of Tregs across autoimmune and allergic diseases remains notably sparse, with most studies addressing these conditions in isolation rather than exploring integrated mechanisms, such as overlapping genetic predispositions (e.g., FoxP3 polymorphisms) or environmental modulators that influence Treg stability in both contexts. This knowledge gap underscores a critical opportunity for deeper investigation, as elucidating common Treg pathways could unveil novel insights into immune homeostasis and disease interconnection.

This review explores the immunobiology of Tregs, emphasizing their shared mechanisms in autoimmune and allergic diseases, and discusses emerging therapeutic avenues to harness Tregs for disease modulation. By bridging the research void on Treg functions in autoimmune and allergic diseases, this review aims to propel advancements in targeted Treg-based therapies, fostering innovative strategies that could simultaneously address the shared immunoregulatory deficits and improve clinical outcomes across these interrelated spectra of immune pathology.

## 2 Immunobiology of Tregs

Tregs represent a specialized lineage of CD4^+^ T cells indispensable for maintaining immune homeostasis and peripheral self-tolerance, thereby preventing autoimmune and allergic pathologies. Their identity and potent immunosuppressive functions are governed by the master transcription factor FoxP3. Phenotypically, Tregs are characterized by high-level expression of the interleukin-2 receptor α-chain (CD25) and low-level expression of the IL-7 receptor α-chain (CD127), a profile that reflects their dependence on IL-2 for survival and lineage stability.

**Figure 1:**
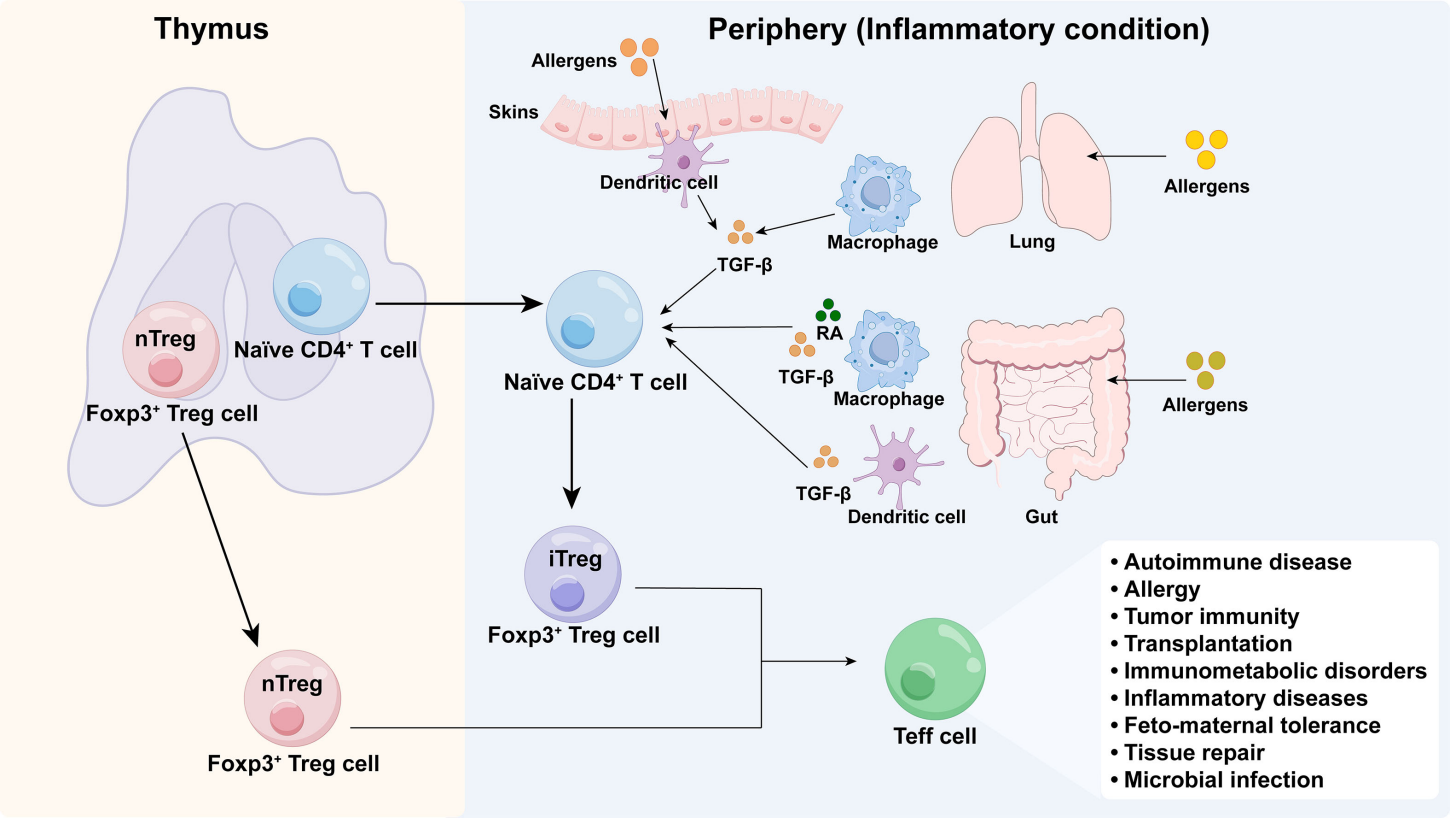
Origin and development of Tregs. Treg cells originate in the thymus, known as natural Tregs (nTregs), or develop in the periphery from naive CD4^+^ T cells into induced Tregs (iTregs). Tolerance to self-antigens is mediated by nTregs, whereas tolerance at mucosal surfaces is maintained by iTregs that develop in peripheral tissues. Stimulation with TGF-β and retinoic acid, as well as exposure to food allergens, promote differentiation of iTregs in the gut. In the lungs, iTregs are activated by airborne allergens and by TGF-β and retinoic acid released by alveolar macrophages. iTregs in the skin are activated by contact with skin allergens and by TGF-β produced by dendritic cells (DCs).

The Treg compartment is broadly divided into two major subsets based on developmental origin: thymic Tregs (tTregs), which arise in the thymus and are also known as natural Tregs (nTregs), and peripherally derived Tregs (pTregs), which differentiate from naïve CD4^+^ T cells in extrathymic tissues (11–14). As depicted in **Figure 1**, these subsets have distinct yet complementary roles. tTregs are crucial for enforcing central tolerance to self-antigens. In contrast, pTregs primarily establish tissue residency, where they are pivotal for orchestrating organ-specific homeostasis and modulating local immune responses to non-self antigens, such as commensal microbiota and allergens (11, 12). For instance, pTreg differentiation in the intestine and lungs can be driven by microbial metabolites or by cytokines like transforming growth factor-β (TGF-β) and retinoic acid (15, 16). Intestinal pTregs, in particular, provide critical protection against food allergies (17, 18). A key distinction between these subsets lies in their T cell receptor (TCR) repertoires; pTregs exhibit greater diversity, enabling them to recognize a broader array of foreign antigens and effectively engage with peripheral pathogens and allergens (19).

**Figure 2:**
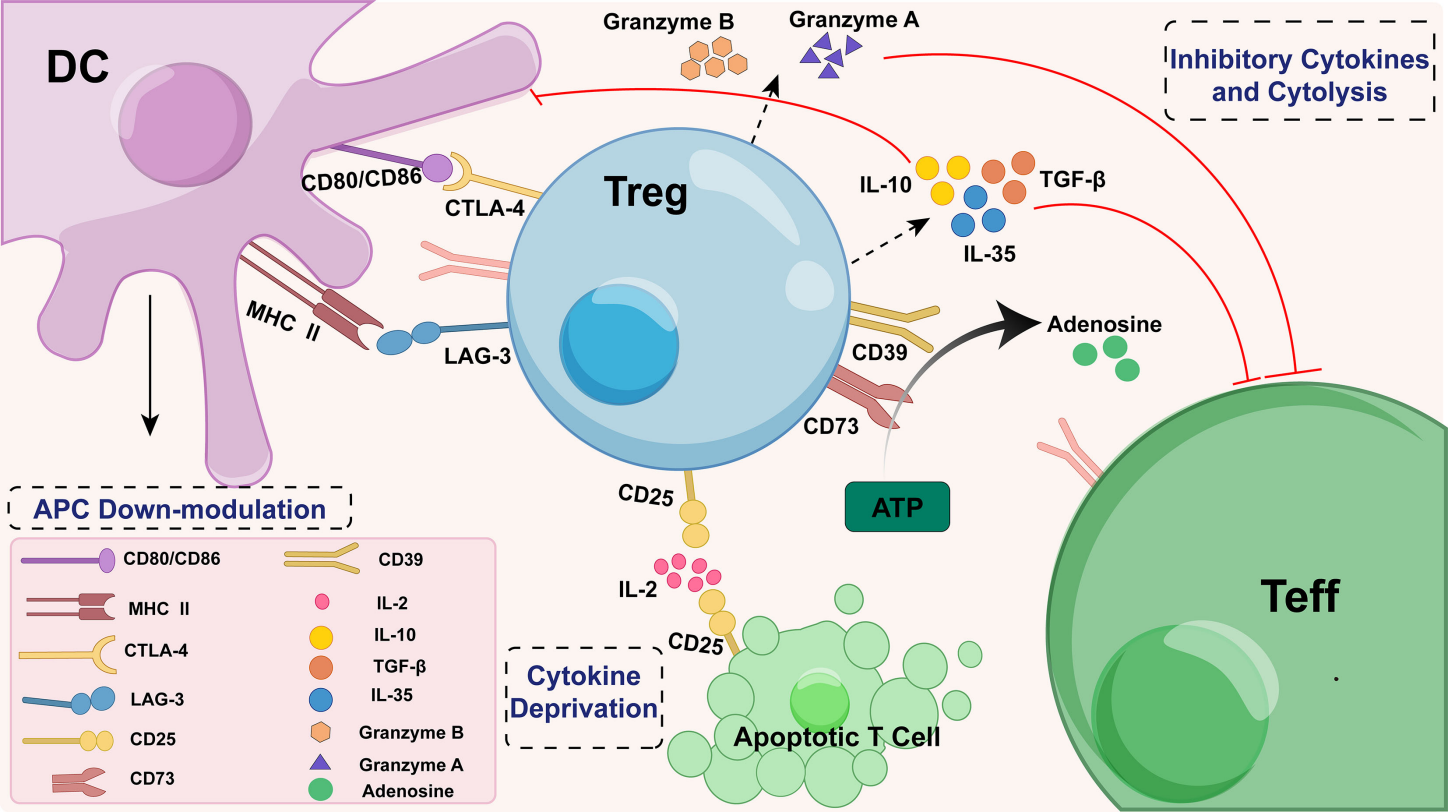
Treg cells suppress immune responses through multiple mechanisms. Tregs regulate both innate and adaptive immune responses through a variety of pathways, such as cytolysis (granzyme), metabolic disruption (CD25, CD39, and CD73), cell-to-cell contact (CTLA-4 and LAG-3), and secretion of inhibitory cytokines (TGF-β, IL-10, and IL-35). ATP, adenosine triphosphate; CTLA-4, cytotoxic T-lymphocyte-associated protein 4; DC, dendritic cell; LAG-3, lymphocyte-activation gene 3; Teff, effector T cell; TGF-β, transforming growth factor β; Treg, regulatory T cell.

Irrespective of their origin, Tregs suppress effector T cell (Teff) responses through a sophisticated and multifaceted array of mechanisms, encompassing both soluble mediators and direct cell-cell interactions (**Figure 2**). Key soluble factors secreted by Tregs include classical immunosuppressive cytokines such as interleukin-10 (IL-10), which inhibits Teff activation, as well as TGF-β and IL-35, which promote Treg differentiation and function. Additionally, Tregs can induce Teff apoptosis through the release of cytotoxic molecules such as granzymes A and B (20–23); intriguingly, granzyme B-expressing Tregs may themselves be more susceptible to apoptosis, potentially serving as a self-regulatory feedback mechanism to prevent excessive tissue damage (24). Cell-cell interactions, meanwhile, engage an array of immune checkpoints, encompassing cytotoxic T lymphocyte antigen 4 (CTLA-4), lymphocyte activation gene 3 (LAG-3), CD73, CD39, and the interleukin-33 receptor (ST2) (25), as depicted in **Figure 2**. The transcription factor Helios bolsters Treg suppressive efficacy and resilience, especially under inflammatory duress (26, 27). Mechanistically, CTLA-4 on Tregs ligates B7 molecules (CD80/CD86) on antigen-presenting cells (APCs) and Teff cells, thereby dampening Teff proliferation and activation (28). LAG-3 engages major histocompatibility complex class II on APCs to propagate inhibitory signals (29, 30). Tregs also harness membrane-bound ectonucleotidases CD39 and CD73, which catalyze the sequential degradation of extracellular adenosine triphosphate (ATP) to adenosine, a potent immunosuppressant (31). Adenosine ligation to Teff receptors curtails their expansion and attenuates proinflammatory cytokine elaboration; however, adenosine accrual exerts nuanced repercussions on Tregs themselves, with protracted or elevated exposure potentially eroding their suppressive vigor (31). Under inflammatory stress, the transcription factor Helios enhances Treg stability and suppressive capacity (32), while ST2, the receptor for IL-33, acts as an “activation sensor” on tissue-resident Tregs, responding to local inflammatory signals to augment their function (33).

The clinical and physiological import of this suppressive versatility crystallizes in the exquisite equilibrium Tregs uphold with diverse Teff subsets, whose perturbation frequently precipitates immune pathologies. Autoimmune conditions, for example, are often propelled by Th1-mediated surges in IL-2 and interferon-γ (IFN-γ), whereas allergic manifestations are chiefly sustained by Th2-driven IL-4 and IL-5 (34). Nowhere is this interplay more salient than in the Treg–Th17 axis, where both lineages derive from shared naïve CD4^+^ progenitors, their trajectories sculpted by the prevailing cytokine milieu. In disorders like inflammatory bowel disease (IBD), a proinflammatory niche skews differentiation toward pathogenic Th17 cells, curtailing Treg emergence and eroding the homeostatic Treg/Th17 balance. This disequilibrium is typified by diminished Treg/Th17 ratios in circulation and lesional tissues, culminating in the hallmark chronic inflammation of such ailments (35).

## 3 Plasticity and epigenetic regulation of regulatory T cells

Tregs exhibit remarkable plasticity, enabling them to adapt their cytokine and chemokine profiles to modulate inflammatory responses through interactions with diverse immune cell subsets. This plasticity allows Tregs to tailor their suppressive functions to specific immunological contexts. For instance, in the Peyer’s patches of the murine intestine, interleukin-6 (IL-6) and IL-21 induce Tregs to differentiate into follicular regulatory T cells (Tfr), which resemble follicular T helper (Tfh) cells (36–38). These Tfr cells migrate to germinal centers, where they promote germinal center formation while suppressing Tfh-driven B cell activation and antibody production (39–41). Similarly, Tregs co-expressing the T-box transcription factor T-bet alongside FoxP3 give rise to Th1-like Tregs, which secrete interferon-γ (IFN-γ) to curb excessive type 2 immune responses in allergic conditions while retaining their suppressive capacity (42–45). The plasticity of Tregs is particularly critical in autoimmune diseases, where Tregs acquire specialized properties to restrain specific T helper (Th) cell subsets (46). However, aberrant Treg plasticity can contribute to immune dysregulation. For example, in type 1 diabetes (T1D), an elevated frequency of IFN-γ^+^FoxP3^+^ (Th1-like) Tregs has been observed in peripheral blood, accompanied by diminished suppressive function and a proinflammatory phenotype (**Table 1**) (47). In chronic inflammatory settings, such as allergic diseases, Tregs can shift from a suppressive to a proinflammatory state. Gut-resident Tregs expressing RAR-related orphan receptor gamma t (RORγt), termed Th17-like Tregs, typically mitigate food allergies. However, in the pulmonary microenvironment, these cells may paradoxically exacerbate allergic asthma by producing IL-17 (48, 49, 51, 53). Additionally, Tregs co-expressing FoxP3, GATA-binding protein 3 (GATA3), and the chemotactic receptor-homolog expressed on Th2 cells (CRTH2) recruit type 2 innate lymphoid cells (ILC2s) to the lungs, where the secretion of IL-4, IL-5, and IL-13 by these Th2-like Tregs aggravates allergic asthma and food allergies (50, 51, 54–56). Prolonged exposure to chronic inflammation can lead to the loss of FoxP3 expression, resulting in the emergence of ex-Tregs—former Tregs that relinquish their suppressive function and adopt a conventional effector T cell phenotype, thereby contributing to pathogenic immune responses and amplifying allergic inflammation (52).

**Table 1:** Treg subtypes.

Subtype	Cellular markers	Related diseases	Role in the immune response	References
Follicular Tregs (Tfr)	FoxP3^+^CD25^+^CXCR5^+^BCL6^+^PD1^+^CTLA4^+^ ICOS^+^	Rheumatoid arthritis, allergic asthma, allergic rhinitis, etc.	Tfr cells enter germinal centers to promote their formation and suppress Tfh-driven B cell responses.	(36, 39)
Th1-like Tregs	FoxP3^+^CD25^+^CD127^low^ T-bet^+^ CCR5^+^ CXCR3^+^	Rheumatoid arthritis, multiple sclerosis, allergic asthma, allergic rhinitis, etc.	Elevated IFNγ secretion may contribute to suppressing excessive Type 2 responses. Decreased inhibitory function in autoimmune diseases.	(42, 47)
Th17-like Tregs	FoxP3^+^CD25^low^ CD127^low^ RORγt^+^ CCR6^+^	Allergic asthma, rheumatoid arthritis, food allergies, etc.	Enhances Th17 responses in the lungs, exacerbating allergic asthma and rheumatoid arthritis, but suppresses food allergies in the gut.	(48, 49)
Th2­like Tregs	FoxP3^+^ CD25^+^ CD127^low^ GATA3^+^ CCR4^+^	Allergic asthma, food allergies, rheumatoid arthritis, tregs in systemic lupus erythematosus, etc.	IL-4, IL-5, and IL-13 expression exacerbates both allergic asthma and food allergy.	(50, 51)
exTregs	FoxP3^low^ CD25^−^ CD127^+^	Allergic inflammation, etc.	The transition from a FoxP3^+^ suppressive cell to an inflammatory effector T cell exacerbates allergic inflammation.	(52)

Epigenetic mechanisms, including DNA methylation and histone modifications, are pivotal in governing Treg plasticity at key genomic loci. Natural Tregs (nTregs) are distinguished from induced Tregs (iTregs) by pronounced DNA hypomethylation at the FoxP3 promoter and enhancer regions, notably the Treg-specific demethylated region (TSDR), also known as conserved noncoding sequence 2 (CNS2) (57, 58). The maintenance of DNA methylation by DNA methyltransferase 1 (DNMT1) and Ten-Eleven Translocation (TET) enzymes is critical for stabilizing FoxP3 expression in nTregs within the thymus; disruption of these enzymes leads to reduced Treg numbers and compromised suppressive function (59). Thus, a characteristic hypomethylation pattern, coupled with sustained methylation maintenance, is essential for preserving nTreg lineage identity and functionality. Beyond methylation, the FoxP3 locus undergoes histone acetylation during Treg development, with histone acetyltransferases (HATs) promoting stable FoxP3 expression (60, 61). Chronic inflammatory environments potently drive epigenetic alterations, directly influencing Treg plasticity. Loss of TET or HAT activity, triggered by infection or metabolic shifts, can precipitate FoxP3 downregulation, prompting Tregs to adopt a Th17-like phenotype (61). Sustained IL-6 signaling, for instance, enhances DNMT1-mediated DNA methylation and histone deacetylase (HDAC) activity, further promoting FoxP3 loss (62). The extensive epigenetic regulation involved in Treg plasticity presents promising therapeutic opportunities, and targeting these epigenetic mechanisms may modulate Treg function in allergic diseases.

## 4 Regulatory T cells in autoimmune and allergic diseases

### 4.1 Regulatory T cells in autoimmune diseases

**Figure 3:**
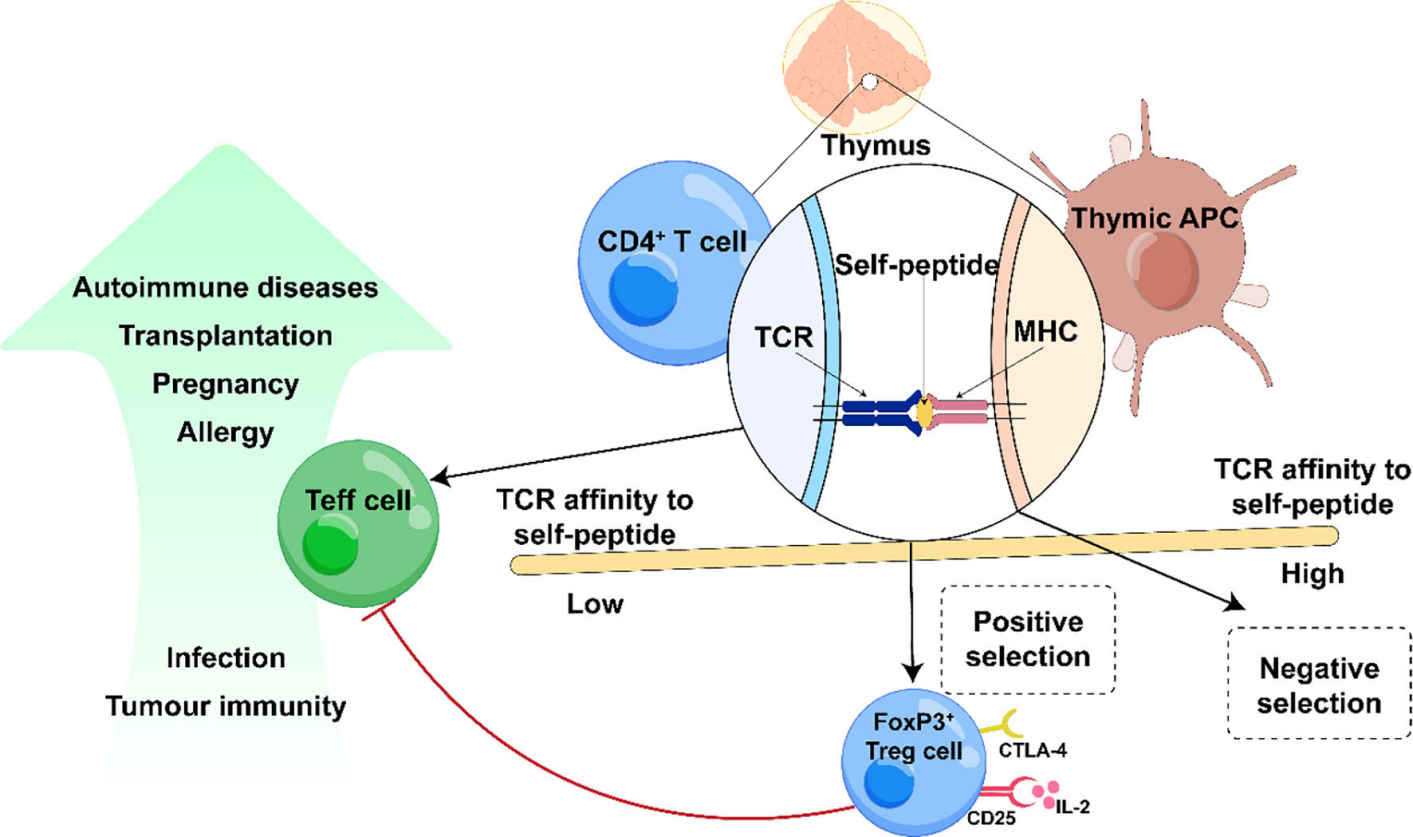
Role of regulatory T cells (Tregs) in autoimmune diseases therapy. Negative selection occurs in the medulla when the TCR of a thymocyte binds with high affinity to a peptide-MHC ligand on medullary thymic epithelial cells, resulting in a self-reactive and subsequent apoptotic cell death. As this process is not always effective, some self-reactive T cells evade elimination and enter the periphery possibly causing autoimmune diseases. High-affinity tissue-restricted binding of MHCII/TCR and subsequent IL-2 signaling leads to upregulation of FoxP3 and CD25. Low-affinity binding results in naive CD4^+^T cells. These naive CD4^+^ T cells may develop in the periphery to iTregs. Tregs play a role in suppressing immune responses directed against both self and non-self-antigens.

Regulatory T cells (Tregs) are essential for maintaining immune tolerance by balancing responses to foreign and self-antigens and preventing excessive inflammation that would otherwise cause tissue damage or fatal immunopathology (63). The critical role of the transcription factor FoxP3 in Treg development and function was first demonstrated in scurfy mice and later in patients with immune dysregulation, polyendocrinopathy, enteropathy, X-linked (IPEX) syndrome, in which loss-of-function mutations in FoxP3 result in a profound deficiency of functional Tregs, leading to uncontrolled effector T-cell (Teff) activation, multiorgan inflammation, autoantibody production, and early mortality (see **Figure 3**) (64, 65). Similar immunodysregulation occurs with defects in other key Treg-associated molecules, such as the IL-2 receptor α-chain (CD25) and cytotoxic T-lymphocyte–associated protein 4 (CTLA-4), with CD25 mutations causing severe autoimmunity and CTLA-4 haploinsufficiency predisposing individuals to a broad spectrum of autoimmune disorders (66, 67). The indispensable role of Tregs in restraining autoimmunity has been further validated in classical experiments using immunodeficient nude mice: reconstitution with CD4^+^ T cells depleted of the CD25^+^ subset induces various organ-specific autoimmune diseases, whereas transfer of the CD4^+^CD25^+^ fraction prevents disease onset (68–70).

Collectively, these findings establish Tregs as central regulators of immune homeostasis and underscore the inadequacy of current autoimmune disease treatments, which remain largely palliative and rely on non-specific systemic immunosuppression rather than correction of underlying immune dysregulation (71). Advances in mechanistic understanding have prompted the development of more targeted therapeutic strategies—such as autoantigen-specific immunomodulation, Treg adoptive cell therapy, and low-dose IL-2 administration—several of which are now being actively evaluated in clinical trials as promising approaches for restoring immune tolerance in autoimmune diseases (72, 73).

#### 4.1.1 Regulatory T cells in rheumatoid arthritis

Rheumatoid arthritis (RA) is a prevalent systemic chronic inflammatory disorder marked by symmetric polyarthritis, which may lead to bone and cartilage erosion and ultimately result in disability (74). Notably, a meta-analysis by Morita et al. (75) revealed that while peripheral blood Treg counts are reduced in RA patients, their abundance is increased in synovial fluid. Nevertheless, these results remain controversial, attributed to the intrinsic heterogeneity of RA and the absence of universally accepted Treg phenotypic markers. Additionally, the transient upregulation of FoxP3 and CD25 following T cell activation in the human immune system complicates the interpretation of data in autoimmune diseases with enhanced T cell activation, highlighting the need for cautious analysis of such findings.

Parallel to observations in RA, Tregs from patients with juvenile idiopathic arthritis (JIA) display features of immune dysregulation, including reduced FoxP3 stability, downregulated CD25 expression (76), altered cytokine and chemokine secretion (77), and diminished responsiveness to IL-2 (76). Notably, *in vitro* studies have shown that Tregs isolated from the peripheral blood or synovial fluid of JIA patients can recover their suppressive capacity once removed from the joint microenvironment (76). This finding suggests that the impaired Treg function observed in the joints of RA and JIA patients is likely not caused by an inherent defect in the Tregs themselves, but rather by the local inflammatory milieu (78). Proinflammatory cytokines—most prominently IL-6—play a pivotal role in inducing Treg instability and fueling inflammatory responses in arthritic conditions. In IL-6-mediated arthritis, CD4^+^ CD25^low^ FoxP3^+^ T cells are prone to losing FoxP3 expression (thus becoming ex-FoxP3 cells) and differentiating into pathogenic Th17 cells, which proliferate within inflamed joints (79). This mechanism underscores how the inflammatory microenvironment can compromise Treg function in arthritic diseases.

Over the past decade, mechanistic studies have emphasized that qualitative defects—rather than absolute Treg counts—better explain regulatory failure in RA (80, 81) Synovial Tregs often show altered expression of canonical markers (FOXP3, CTLA-4, CD25, Helios) and exhibit “fragilization,” a state marked by reduced suppressive capacity and enhanced susceptibility to pro-inflammatory reprogramming. Reduced expression of Ikaros family transcription factors (Helios, Aiolos, Eos) and incomplete demethylation of the FOXP3 TSDR correlate with diminished lineage stability and heightened disease activity, suggesting potential applications as biomarkers for disease stratification (27, 82, 83).

Taken together, existing findings demonstrate that RA is defined not by a mere numerical shortage of Tregs but rather by substantial impairments in Treg stability and functional capacity, orchestrated by chronic inflammation and local tissue-derived signals. Therapeutic strategies targeting Tregs hold considerable potential. In a murine collagen-induced arthritis model, adoptive transfer of Tregs markedly mitigated joint destruction through the suppression of T and B cell activity, as well as the inhibition of osteoclast-driven bone resorption (84). These findings underscore the potential of Tregs as a feasible therapeutic approach for autoimmune conditions marked by heightened proinflammatory cytokine production.

#### 4.1.2 Regulatory T cells in systemic lupus erythematosus

Systemic lupus erythematosus (SLE) is defined as a chronic systemic autoimmune condition marked by diverse clinical features, the formation of autoantibodies, and a fundamental failure of immunological self-tolerance (85). A decrease in circulating Treg numbers is often observed during active SLE, and this numerical decline frequently correlates with the clinical severity of the disease (86). Evidence from murine lupus models suggests that therapeutic strategies focused on Tregs hold significant promise for improving the management of SLE (68). For instance, when lupus-prone (SWRxZNB)F1 mice received a subcutaneous administration of 1 μg of nucleosomal histone peptide autoepitopes, they successfully generated potent CD4^+^CD25^+^ and CD8^+^ Tregs. Crucially, these Tregs effectively reduced lupus-associated autoimmunity without inducing systemic immunosuppression, allergic responses, or anaphylaxis. Following adoptive transfer, these cells demonstrated the capacity to halt the migration and accumulation of pathogenic autoimmune cells in critical target sites, specifically the kidneys of lupus nephritis-susceptible mice, alongside suppressing autoantibody production and autoantigen recognition (87). Consistent with animal studies, multiple human investigations have documented a reduced prevalence of Tregs in SLE patients (86, 88). Moreover, Tregs isolated from individuals with active SLE exhibit a compromised ability to suppress the proliferation and cytokine secretion of CD4^+^ T effector cells *in vitro*. This functional deficit is linked to lower expression levels of both FoxP3 mRNA and protein (89).

#### 4.1.3 Regulatory T cells in primary Sjogren’s syndrome

Primary Sjögren’s syndrome (pSS) is recognized as a systemic autoimmune disorder fundamentally defined by the infiltration of lymphocytes into the salivary and lacrimal glands (90). Though exocrine glands are the principal targets, pSS can affect various other organ systems. T cell-driven mechanisms are widely considered central to pSS pathogenesis, ultimately resulting in B cell overactivity. The activation of T cells promotes the loss of self-tolerance and facilitates the release of pro-inflammatory cytokines—including IFN-γ, IL-17, and IL-21—which drive local inflammation (90). The exact contribution of Tregs to pSS pathophysiology remains unsettled, given that published studies report conflicting results, describing Treg frequencies as normal, elevated, or diminished (68). Furthermore, the localization and presence of Tregs within the salivary glands, the primary target organ, are also points of debate. Certain investigations have established a positive correlation between the degree of CD4^+^FoxP3^+^ T cell infiltration in lymphocytic sialadenitis and biopsy severity scores. Conversely, other reports have indicated a marked scarcity or absence of Tregs within the inflamed glands, even when the count of circulating Tregs remains stable (68).

#### 4.1.4 Regulatory T cells in experimental autoimmune encephalomyelitis, multiple sclerosis, and Parkinson’s disease

Tregs are instrumental in managing the autoimmune response in experimental autoimmune encephalomyelitis (EAE), the most common animal model for multiple sclerosis (MS). MS is characterized by demyelination and inflammation within the central nervous system (CNS), alongside a marked infiltration of lymphocytes (91). Studies in mice demonstrate that intentional T cell depletion can provoke spontaneous autoimmune disease, while conversely, boosting Treg activity can mitigate or prevent various EAE manifestations (92). Adoptive transfer of Tregs has been shown to substantially reduce the clinical severity of EAE, suggesting that CD4^+^CD25^+^ Tregs suppress both CNS inflammation and antigen-specific autoreactive immunity during the active phase of the disease (93). *In vitro*, data confirm that Tregs are potent inhibitors of CD4^+^ T cell-dependent Th1 cytokine production, particularly in response to myelin oligodendrocyte glycoprotein (MOG) (94). Notably, in MOG-induced EAE models, induced Tregs (iTregs) demonstrate comparable efficacy to natural FoxP3^+^ Tregs in halting disease progression (95).

One promising experimental therapeutic strategy for EAE involves a *Salmonella* vaccine engineered to express the anti-inflammatory colonization factor antigen 1 (*Salmonella* CFA/1) (96). This vaccine was shown to elevate the population of CD4^+^CD25^+^FoxP3^+^ Tregs and successfully inhibited EAE onset in SJL mice. The Tregs generated by this specific vaccine were even more effective in EAE prevention compared to naive Tregs or those induced by the standard *Salmonella* vector alone. Other experimental treatments also offer hope for EAE. For instance, both the transfer of CD4^+^CD25^+^ T cells and myelin basic protein (MBP)-specific receptor-modified T cells have been utilized to prevent and treat MBP-induced EAE. Furthermore, the heparin-binding growth factor midkine (MK), whose levels are markedly increased in the spinal cord during EAE development, plays roles in inflammation, tissue repair, and oncogenesis (97, 98). Deficiency of MK was found to alleviate MOG-induced EAE by increasing Treg frequency in peripheral lymph nodes and simultaneously suppressing autoreactive Th1 and Th17 cells. Given that MK is a potent inhibitor of Treg proliferation, blocking it with RNA aptamers represents a potential new treatment avenue for autoimmune diseases, as its neutralization effectively expands Treg populations and lessens EAE symptoms (99).

Parkinson’s disease (PD), the second most prevalent neurodegenerative condition after Alzheimer’s disease, involves the progressive loss of dopaminergic neurons in the substantia nigra and their projections into the caudate-putamen (100). The neuroprotective effects demonstrated by Tregs in animal models of PD underscore the potential for Treg-based therapies to slow neurodegeneration and preserve dopaminergic neurons (101).

#### 4.1.5 Regulatory T cells in inflammatory bowel disease

Inflammatory bowel disease (IBD), a category encompassing Crohn’s disease and ulcerative colitis, impacts an estimated 0.3% of individuals in Western countries (102). Tregs have been shown to be essential for both preventing and resolving colitis, particularly in animal models of gut inflammation (103). The therapeutic effect of CD4^+^CD25^+^ Tregs in colitis involves the restoration of normal intestinal structure and a decrease in leukocyte infiltration within the intestinal lamina propria (104). A substantial number of Tregs have been found in both the inflammatory lesions and the mesenteric lymph nodes (105). Their ability to curb the accumulation of effector cells in the colon suggests they may be capable of arresting the progression of colitis (106). In patients with active Crohn’s disease, while FoxP3^+^CD4^+^ Treg cells are often diminished in the peripheral circulation, their concentration is typically increased within mucosal lymphoid tissues, including the lamina propria and mesenteric lymph nodes (107). These cells frequently congregate in sites of active inflammation, such as granulomas. An exciting finding in a chronic T cell-dependent colitis model involved the parenteral delivery of filamentous hemagglutinin (FHA) from *Bordetella pertussis* to severely immunocompromised mice. This approach successfully lowered the counts of Th1 cells and pro-inflammatory cytokines, activated Tregs, and reduced disease activity (108). This result is particularly compelling because FHA appears to be a viable candidate for clinical evaluation in patients with Crohn’s disease.

#### 4.1.6 Regulatory T cells in autoimmune diabetes

Type 1 diabetes (T1D), also known as insulin-dependent diabetes mellitus, is an autoimmune disorder resulting from the immune system’s destructive targeting of pancreatic β cells. The inbred non-obese diabetic (NOD) mouse model, which spontaneously develops an autoimmune diabetes highly analogous to human T1D, is a critical tool for mechanistic studies (109). In NOD mice, the dynamic balance between diabetogenic T cells and Tregs is crucial for regulating diabetes progression, with disease onset often linked to a progressive loss of Treg function (110, 111). Adoptingly transferring islet-specific Tregs can prevent both established and early insulitis, conferring protection against spontaneous diabetes (112). Additionally, polyclonal Tregs or *adaptive* Tregs derived from normal CD4^+^ populations are capable of reversing the disease shortly after diagnosis. These cells mature into FoxP3^+^CD25^-^ memory Tregs, which offer durable protection against relapse (112). A noteworthy recent investigation showed that a small count of pancreatic islet-antigen-specific Tregs was substantially more efficacious at preventing and treating diabetes in NOD mice than a far greater number of polyclonal Tregs (113). Moreover, autoimmune diabetes can be prevented in Treg-deficient NOD mice by culturing and utilizing Tregs that are specific to islet peptide mimics (114, 115). These observations provide support for the concept that naturally existing autoantigen-specific Tregs could be harnessed for the therapy of organ-specific autoimmunity. As the disease advances, Tregs selectively traffic to the pancreas, where they inhibit T effector cell activity to manage the later phases of diabetogenesis. Nevertheless, Treg-mediated control is compromised with age, as Tregs show reduced proliferation within the pancreas and decreased functional capacity, raising susceptibility to the disease (116). Furthermore, an association has been established between Coxsackievirus B_4_ (CB_4_) infections and T1D induction (117). Interestingly, Tregs produced during CB4 infection under the influence of TGF-β demonstrated an ability to protect against T1D development without impairing the necessary antiviral immunity. This suggests a potential avenue for infection-mediated immune regulation in preventing insulin resistance.

#### 4.1.7 Regulatory T cells in chronic kidney disease

Glomerulonephritis is a major contributor to chronic kidney disease (CKD) and end-stage renal disease, and although some kidney injuries are non-immune, it is primarily viewed as an immune-mediated condition (118). Tregs possess a substantial ability to modulate both the extent of tissue injury and subsequent repair mechanisms in various renal disorders (119). Investigations into the therapeutic potential of these cells in animal models indicate that treatment approaches focused on Tregs could be advantageous for both preventing and managing kidney disease in humans (120, 121).

Tregs have been demonstrated to be powerful suppressors of anti-glomerular basement membrane (anti-GBM) glomerulonephritis, a severe autoimmune disease affecting the kidney (122). Tracing experiments using GFP-labeled Tregs revealed that, contrary to expectation, these cells mainly accumulated in the spleen and lymph nodes draining the kidney, rather than directly infiltrating the kidneys of mice with nephritis (120). Interestingly, Treg administration did not reduce the formation of immune complexes in the glomeruli. Instead, they lessened end-organ damage by inhibiting the activation of immune cells within the adjacent lymph nodes. In the context of Goodpasture’s syndrome, a human autoimmune disorder, T cells specific for collagen exhibited an inflammatory profile during active phases but transitioned to a regulatory function during remission, which highlights the central role Tregs play in resolving this autoimmune response (123). Furthermore, in the adriamycin nephropathy mouse model of chronic proteinuric renal disease, naive T cells were genetically modified by retroviral transduction with the FoxP3 gene to produce FoxP3-transduced Tregs. These modified cells displayed a regulatory profile, successfully suppressed the *in vitro* proliferation of CD4^+^CD25^+^ T cells, and *in vivo* reduced both glomerular and interstitial damage, thereby preserving renal structure and function (121). Nevertheless, obstacles related to vector delivery have made gene therapy aimed at the kidney challenging (124). Although the strategy of utilizing Tregs to impede or stop the advancement of renal disease is attractive, comprehensive, disease-specific research and meticulously planned clinical trials are mandatory before these treatments can be routinely applied to human patients.

#### 4.1.8 Regulatory T cells in autoimmune gastritis and acquired aplastic anemia

Autoimmune gastritis (AIG) is a spontaneously occurring and rare animal model for organ-specific autoimmunity. Its primary target antigen is the gastric parietal cell proton pump, H-K-ATPase (125). AIG also functions as a mouse model for human pernicious anemia, characterized by T and B cell reactivity against H-K-ATPase. A study by Khalilollah et al. established that T effector (Teff) cells, specifically Th1, Th2, and Th17 subsets, drive AIG pathology, with each subset generating unique histological patterns of tissue injury. Notably, Th17 cells were identified as the subset causing the most widespread gastric destruction via cellular infiltration (126). In contrast, the concurrent transfer of naturally occurring polyclonal Tregs was effective at completely preventing AIG development. Although Tregs were very successful in mitigating Th1 and Th2 cell-mediated pathology, their efficacy in controlling Th17-induced AIG was limited to the disease’s initial stages (127). Turning to acquired aplastic anemia, contemporary evidence suggests that patients with the condition have significantly reduced circulating levels of Tregs (128). In murine models, Treg infusion has been shown to decelerate disease progression. Further human research indicated that virtually all aplastic anemia patients had a lower frequency of Tregs at the time of diagnosis, coupled with significantly diminished expression of FoxP3 and the critical immune-regulatory transcription factor, NFAT1 protein (129).

#### 4.1.9 Regulatory T cells in Hashimoto’s thyroiditis

Hashimoto’s thyroiditis (HT) is an organ-specific autoimmune condition defined by the infiltration of lymphocytes into the thyroid gland, which culminates in the destruction of follicles (130). Experimental Autoimmune Thyroiditis (EAT) is a validated mouse model used to investigate HT (113). Studies have demonstrated that granulocyte-macrophage colony-stimulating factor (GM-CSF) fosters the generation of both IL-10-producing Tregs and semi-mature dendritic cells (DCs). This evidence proposes GM-CSF as a potential therapeutic agent for EAT and for other autoimmune diseases sharing similar underlying pathology. A crucial finding is that IL-10 directly suppresses mouse thyroglobulin-specific T effector cells, underlining its vital function in controlling the disease in mice treated with GM-CSF (131). Furthermore, semi-mature DCs loaded with thyroglobulin exhibit tolerogenic characteristics that effectively stop EAT progression by facilitating the proliferation of thyroglobulin-specific Tregs. Apart from their function in thyroid autoimmunity, Tregs have also been suggested to play a role in the transition of hyperthyroid Graves’ disease into HT and hypothyroidism (132).

#### 4.1.10 Regulatory T cells in psoriasis

Psoriasis is a systemic inflammatory disorder influenced by a combination of environmental factors and genetic susceptibility (133). In an immunocompetent state, Tregs rigorously control the secretion of Type 1 and Th17 cytokines, both of which are central pathogenic drivers of psoriasis. By inhibiting these pro-inflammatory cell types, Tregs maintain immunological equilibrium and help guard against autoimmune conditions, including psoriasis and other dermatological issues (134). A failure in Treg-mediated suppressive function is hypothesized to shift the Th17/Treg balance in psoriasis, a shift that has been associated with worsened disease presentation. Nevertheless, the exact molecular basis for Treg dysfunction in psoriasis has yet to be fully elucidated. Despite the widely accepted importance of Tregs for therapeutic approaches in psoriasis, the connection between the cellular concentration of Tregs and the clinical severity of the disease is still a matter of ambiguity (135, 136).

### 4.2 Regulatory T cells in allergic diseases

This section comprehensively examines the distinct roles and functional impairments of Tregs across major allergic conditions, including allergic rhinitis (AR), allergic asthma, food allergy (FA), and atopic dermatitis (AD). It focuses on how alterations in the frequency, phenotypic characteristics, and suppressive function of Tregs contribute to the underlying disease pathology, and further evaluates the therapeutic viability of Treg-based interventions.

#### 4.2.1 Regulatory T cells in allergic rhinitis

Allergic rhinitis (AR) is a pervasive, chronic inflammatory disorder affecting the upper respiratory tract, impacting an estimated 20% to 30% of adults and up to 40% of children across the United States and Europe (137, 138). Its complex pathophysiology involves inflammation of the nasal mucosa, orchestrated by a sophisticated network of immune cells, notably ILC2s, dendritic cells (DCs), Th2 cells, follicular T helper (Tfh) cells, follicular regulatory T (Tfr) cells, and B cells (139, 140).

Tregs are widely recognized for their potent capability to regulate and inhibit allergen-specific immune responses (141). Across numerous studies in AR patients, a consistent finding is the diminished count of circulating FoxP3^+^ Tregs, alongside a reduced *in vitro* ability to suppress Th2-driven responses when compared to healthy controls (142). Concomitantly, decreased FoxP3 expression levels have been observed within the nasal mucosa of AR subjects (143–145), which may correlate with the clinical severity of the condition (146). Genetic research has established links between specific FoxP3 gene polymorphisms and AR, implying that these variations might compromise Treg functionality and heighten susceptibility to allergic reactions (147). Furthermore, an elevated proportion of immunoglobulin-like transcript 3-positive (ILT3+) Tregs, characterized by lower FoxP3 expression and impaired suppressive capacity, has been identified in AR cohorts (148, 149). Patients with AR also exhibit lower levels of suppressive IL-35-producing Tregs (iTr35) and circulating IL-10-producing Tr1 cells (150). Additional reports highlight decreased frequencies of CD8^+^CD25^+^CD137^+^ Tregs and Tfr cells in the nasal mucosa, peripheral blood, and tonsils of individuals with AR (151, 152). Conversely, some data suggest a higher relative abundance of IL-17A-secreting, FoxP3- T cells (potentially indicative of plastic Tregs or Th17-like cells) in the AR population compared to non-allergic controls.

One investigation utilizing a murine AR model detected a lower population of Helios+ Tregs in the nasal mucosa and splenic cells of AR mice relative to controls. This observation proposes that defective Treg suppressive activity facilitates the excessive activation of T helper cells, such as Th2 cells, and subsequently promotes disease onset. The study also determined that approximately 75% of CD25^+^FoxP3^+^ Tregs co-expressed Helios^+^, suggesting that while the FoxP3^+^Helios^+^ subset is a major component, the combined FoxP3^+^CD25^+^ marker captures a larger overall Treg population in both AR and control groups (153). In a separate study, it was demonstrated that Notch2 can directly enhance the transcription of FoxP3, speculating that this mechanism fosters Treg differentiation and function. This, in turn, could inhibit pro-inflammatory and effector T cell responses, leading to a significant mitigation of the allergic inflammatory response characteristic of AR (154). Collectively, the evidence points toward a reduction in both the absolute number and the functional potency of Tregs in AR, which profoundly influences its pathophysiology and clinical course. Consequently, the restoration of normal Treg function is regarded as a pivotal therapeutic objective in the management of AR.

#### 4.2.2 Regulatory T cells in allergic asthma

Allergic asthma is a persistent respiratory condition initiated by allergic hypersensitivity to specific environmental allergens, presenting with hallmark features like airway hyperresponsiveness, elevated immunoglobulin E (IgE) levels, and chronic inflammation of the airways (155). Common sensitizing agents include dust mites, animal dander, pollen, and fungi. The inflammatory process in allergic asthma is primarily a Th2-mediated reaction, engaging both the innate and adaptive immune systems (156). CD4^+^ Th2 cells secrete characteristic cytokines (IL-4, IL-5, IL-9, and IL-13), which collectively drive eosinophil recruitment to the airway wall, induce mucus hypersecretion, and stimulate IgE synthesis by allergen-specific B cells, culminating in the massive degranulation of mast cells and release of inflammatory mediators (157).

Treg dysfunction is a critical factor in asthma pathogenesis, as it disrupts the essential process of immune tolerance. Within the lung environment, Tregs play a role in promoting the differentiation of regulatory B cells and biasing DCs toward a tolerogenic phenotype, which collectively impedes initial sensitization and IgE synthesis upon allergen encounter (141). Pulmonary Treg populations inhibit key effector cells in allergic asthma, including Th2 cells, mast cells, eosinophils, basophils, and ILC2s. This suppression is achieved through soluble mediators such as IL-10, TGF-β, and IL-35, as well as via cell-surface inhibitory molecules like PD-1 and CTLA-4 (158). Early experimental work in mouse models established that the selective depletion of CD4^+^CD25^+^ Tregs exacerbated airway hyperresponsiveness, increased IL-4 and IL-5 production, and heightened the influx of T cells and neutrophils into the airways during allergic asthma (159).

Given the Th2-driven nature of allergic asthma, Tregs, owing to their capacity to inhibit Th2 activation, are fundamentally important. Studies examining Treg counts in asthma, however, have yielded contradictory outcomes. One research group reported a decline in lung Tregs among asthmatic children, which they correlated with suppressed pulmonary Th2 reactivity (160). Conversely, other investigations suggested a trend toward an increased number of Tregs residing in the airways of patients afflicted with moderate to severe asthma, relative to healthy individuals (161). These disparities likely stem from differences in study populations and the methodologies employed for Treg enumeration. Adding further complexity, some evidence suggests that low Treg counts and functional impairments may predispose younger patients (children and young adults) to asthma, whereas the correlation between Tregs and asthma risk or severity appears less pronounced in older patients (162).

Alveolar macrophages and pDCs have been pinpointed as vital cell types that promote FoxP3+ Treg differentiation within the lung microenvironment (153). In individuals with severe asthma, a reduction in the number of FoxP3^+^ Tregs has been documented in both peripheral blood and bronchoalveolar lavage fluid (BALF) samples when compared to healthy subjects (163–167). This numerical decrease is frequently accompanied by a compromised ability of Tregs to chemotax toward lung epithelial cells (165, 168). Additionally, FoxP3^+^ Tregs from these patients exhibit reduced expression of CCR5, suggesting impaired suppressive activity that correlates with worsened lung function (166). Furthermore, Tregs in asthma exhibit elevated expression of CRTH2, a Type 2 receptor for prostaglandin D2, which is associated with asthma control and exacerbations (165). Thus, allergic asthma is characterized by both reduced Treg numbers and altered surface marker expression (e.g., low CCR5 and high CRTH2), collectively pointing to impaired function and a susceptibility to Th2-skewed inflammatory responses (166).

A considerable body of research underscores the profound influence of environmental factors on asthma exacerbation. For instance, exposure to elevated levels of ambient air pollution is a recognized risk factor, potentially mediated by epigenetic modifications affecting Treg function. A study by Prunicki et al. (167) demonstrated distinct patterns of FoxP3 gene methylation in asthmatic subjects exposed to air pollution compared to non-asthmatic controls under similar exposure conditions. More recent studies further link changes in DNA methylation within the FoxP3 promoter region to subsequent impairment of regulatory T cell function (169). Common atmospheric contaminants, including carbon monoxide (CO), nitrogen dioxide (NO_2_), polycyclic aromatic hydrocarbons (PAHs), and particulate matter (PM), induce altered CpG methylation at the FoxP3 locus. This mechanism compromises Treg activity and worsens asthma phenotypes (170). Additional research indicates a strong correlation between exposure to air pollutants in children and FoxP3 methylation levels, associating with Treg dysfunction and increased plasma IgE (170). Moreover, exposure to inhalable particulate matter has been shown to disturb the critical Treg/Th17 balance, aggravating asthma via a mechanism dependent on the aryl hydrocarbon receptor (AHR). Following PM-induced Ahr activation, the Notch ligand JAG1 is expressed, which destabilizes iTregs and promotes allergic airway inflammation. Recent findings identify Notch4 as a relevant Notch receptor on Tregs that is upregulated in circulating Tregs from asthmatic patients in an IL-6-dependent manner, correlating with disease severity (171, 172). Notably, blocking the IL-6 receptor signaling pathway enhances Treg suppressive function and downregulates Notch4 expression, offering a potential therapeutic benefit for patients with severe asthma (173, 174).

In summary, compelling evidence suggests that Tregs are central to the pathogenesis of allergic asthma. The disease pathology is frequently exacerbated by reduced numbers and impaired function of Tregs in the lungs, positioning Tregs as a valuable target for potential therapeutic interventions.

#### 4.2.3 Regulatory T cells in food allergy

Food allergy (FA) constitutes a major global public health concern, with its prevalence escalating rapidly in recent decades. It is defined by predictable IgE-mediated adverse reactions upon consumption of specific foods (175, 176). Surveillance data from the United States indicate that approximately 7.6% of children are affected by FA, with a notable 18% surge in prevalence reported between 1997 and 2007 (175, 176). In the UK, the rate of hospital admissions for FA in young children increased by 6.6% annually between 1998 and 2018 (177).

**Figure 4:**
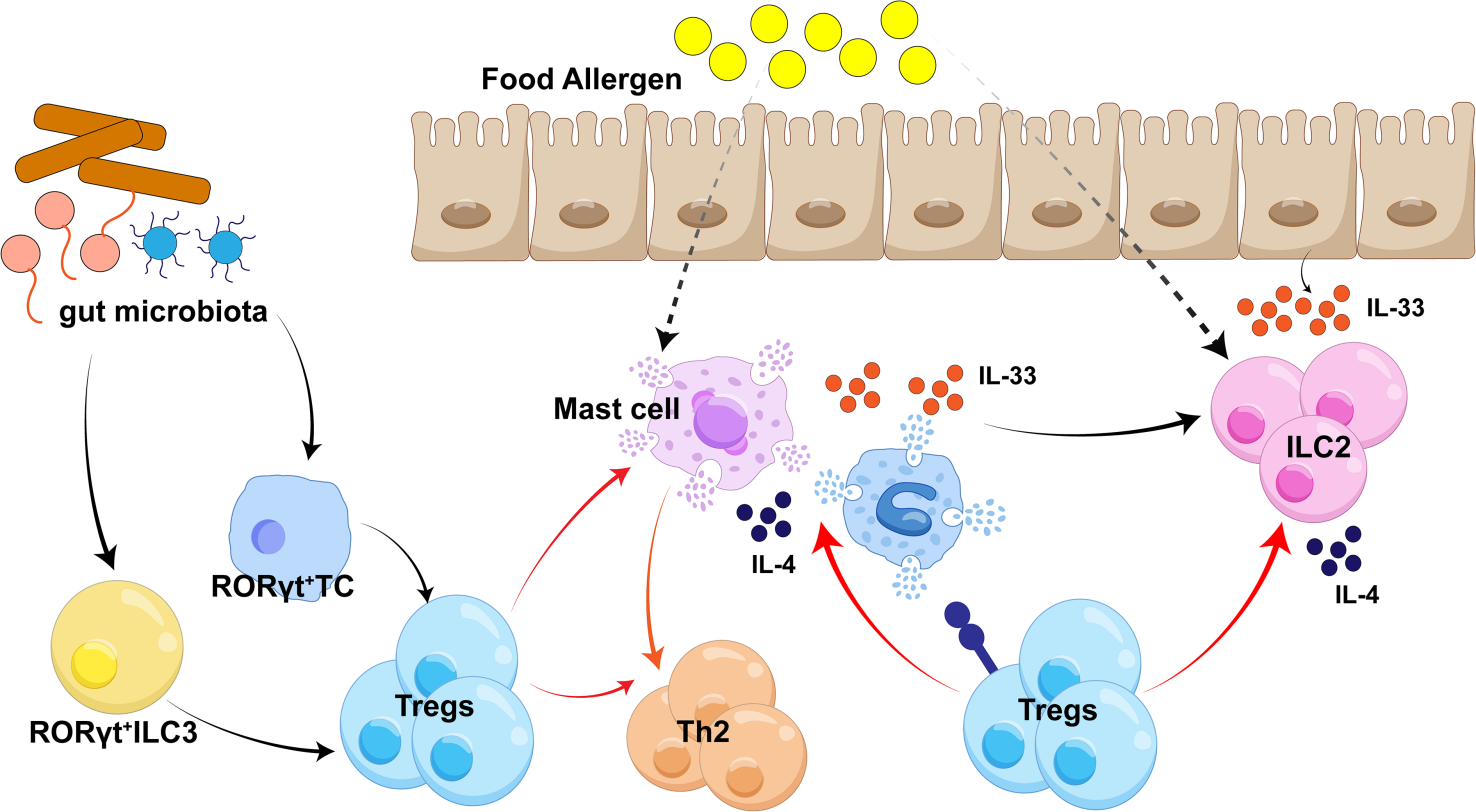
Tregs in food allergy (FA). Treg cells restrain IL-33–induced expansion of ILC2s in the intestinal mucosa, thereby reducing IL-4 production. RORγt^+^ antigen-presenting cells (APCs), including type 3 innate lymphoid cells (ILC3s) and Thetis cells (TCs), sample commensal antigens from the intestinal lumen and promote activation of RORγt^+^ induced Treg (iTreg) cells. These commensal-induced RORγt^+^ Tregs, via a TGF-β1–dependent mechanism, maintain intestinal immune tolerance in food allergy by suppressing mast cell activation and allergen-specific Th2 responses.

FA is thought to emerge from a complex interplay of genetic predisposition, a history of atopy, family history of allergies, shifts in hygiene practices, and the timing and route of food antigen exposure (178). In pediatric populations, the most frequent allergens include peanuts, milk, nuts, and eggs, while adults are more commonly affected by fish, shellfish, nuts, and peanuts (179–181). Oral tolerance, the body’s natural state of immunological unresponsiveness to antigens ingested orally, is a fundamental protective mechanism. Its failure triggers a pathogenic Type 2 immune response, marked by the synthesis of high-affinity IgE antibodies against food antigens (182). Allergen sensitization can occur not only through the gastrointestinal tract but also via the respiratory tract or the skin. Once the epithelial barrier is compromised, antigen-presenting cells (APCs)—such as macrophages and dendritic cells (DCs)—process and present allergens to T cell receptors, initiating a Th2-type immune response and the subsequent generation of antigen-specific IgE (183). This IgE then cross-links with FcεRI receptors on basophils and mast cells, leading to their degranulation and the swift release of inflammatory mediators (175, 184). The resulting rapid symptoms can include urticaria, rash, or gastrointestinal upset. In severe instances, life-threatening complications like anaphylaxis or cardiovascular/respiratory abnormalities may arise (See **Figure 4** for an overview of allergic disease mechanisms) (185, 186).

Tregs are unequivocally established as pivotal in the induction of oral tolerance. The process by which DCs stimulated by food antigens generate Tregs is intricate, encompassing multiple dependent mechanisms, including those reliant on retinoic acid, indoleamine 2,3-dioxygenase (IDO), or TGF-β. The expression of integrin α4β7 and CCR9 on induced Tregs governs their directed homing to the gut (187). Moreover, metabolites derived from the gut microbiota significantly contribute to the initiation of tolerogenic pathways (188–190). As noted, the indispensable role of Tregs in preventing Type 2 cytokine secretion and mast cell degranulation underscores their function in curtailing the progression of allergic reactions (191). Indeed, FA has been linked to compromised functionality and generation of allergen-specific Tregs, as demonstrated in a mouse model with enhanced IL-4 receptor signaling (Il4ra^F709^) (192). Furthermore, the adoptive transfer of Tregs has been shown to prevent anaphylaxis in a murine model of ovalbumin-induced FA (193). Patients diagnosed with FA have consistently been reported to have a lower percentage of circulating Tregs compared to healthy counterparts (194–196). Additionally, the normal age-related increase in CCR6 expression observed on FoxP3^+^ Tregs from healthy individuals was absent in children with food allergies. This deficiency in CCR6 expression may impede Treg migration to peripheral inflammatory sites, thereby hindering tolerance induction (194). In recent years, substantial research has highlighted the influence of epigenetics on oral immune tolerance. Oral immunotherapy (OIT) using peanut protein has been shown to increase the population of antigen-specific Tregs and enhance DNA demethylation at the FoxP3 gene locus (195). These specific epigenetic alterations have been attributed to IDO-expressing DCs isolated from participants undergoing OIT (195). In a murine peanut allergy prevention model, the administration of high (but not low) doses of peanut before sensitization successfully induced tolerance and elevated the percentage of CD4^+^CD25^+^FoxP3^+^ cells in mesenteric lymph nodes (MLN) (196). The study found that tolerized mice exhibited lower methylation levels of the critical regulator FoxP3 compared to mice sensitized to peanut protein (196). Finally, dietary components have been proposed to function as epigenetic regulators, potentially offering a means to restore the dysregulated immune balance associated with food allergies.

The host’s intestinal immune system is continuously challenged by, and interacts with, immunogenic molecules and antigens derived from both ingested food and the intestinal commensal microbiota. In the neonatal period, the gut environment is predominantly colonized by species such as *Lactobacilli* and *Bifidobacteria*. These early residents secrete specific neurotransmitters that stimulate Treg activation early in life, a process crucial for establishing long-term immunological tolerance to dietary antigens (197). The shift toward solid food during weaning promotes the proliferation of the *Clostridium* and *Bacteroidetes* phyla. This microbial transition subsequently drives the robust induction of a specialized Treg subpopulation termed RORγt^+^ Tregs (197). Induced by gut microbiota early in life, RORγt^+^ Tregs persist into adulthood and contribute to tolerance toward food and commensal antigens by suppressing pathogenic Th1, Th2, and Th17-mediated immune responses (198). Notably, MHCII^+^RORγt^+^ antigen-presenting cells (APCs)—distinct from conventional dendritic cells (DCs)—have been identified as key regulators of RORγt+ Treg differentiation (199, 200). These RORγt^+^ APCs comprise type 3 innate lymphoid cells (ILC3s) and a recently characterized cell type called Thetis cells. Both ILC3s and Thetis cells mediate RORγt^+^ Treg induction through TGF-β1 signaling, which relies on an αVβ integrin-dependent mechanism, with Thetis cells exerting a dominant role in early life (see **Figure 4**). Additionally, the development of RORγt^+^ Tregs is guided by immunogenic signals from the microbiota, such as polysaccharides and secondary bile acids (201, 202).

In conclusion, regulatory Tregs are undeniably critical for maintaining immune tolerance to food antigens. Deficiencies in Treg induction or functional competence profoundly impact the onset of allergic responses. As such, targeting Tregs to restore immune tolerance represents a prominent therapeutic strategy for the management of food allergies.

#### 4.2.4 Regulatory T cells in atopic dermatitis

Atopic dermatitis (AD), commonly referred to as eczema, is a widespread inflammatory cutaneous disorder that impacts roughly 2–3% of adults and 10–20% of children (203). Distinguishing features include impaired skin barrier function, aberrant cellular immune responses, and heightened susceptibility to environmental allergens—with Th2 cell overactivation serving as a central driver (204). Key pathological traits of AD lesions encompass the infiltration of activated Th2 cells and eosinophils, alongside the expansion of ILC2s, secretion of IL-4 and IL-13, and increased concentrations of total and allergen-specific IgE (205). These pathological hallmarks correlate directly with disease severity. Notably, Tregs are highly enriched in the skin of both humans and mice, where they play a critical role in regulating allergic inflammation and facilitating tissue repair (206–208).

Skin-resident Tregs are established in early life and contribute to the induction of tolerance toward the cutaneous microbiota (209, 210). A large proportion of skin Tregs express the transcription factor GATA3 (211, 212), which aligns with their role in tissue repair alongside other type 2 immune components, such as ILC2s and Th2 cells. Emerging data indicate that these Tregs also express the retinoic acid receptor-related orphan receptor α (RORα)—a molecule thought to constrain dysregulated type 2 immune reactions within the skin (213). Furthermore, skin Tregs express alarmin receptors, including IL-33R and TSLPR (214, 215). This capacity to sense tissue damage enables Tregs to co-mobilize with the subsequent type 2 immune response, promoting tissue repair through alarmin-induced production of amphiregulin (Areg).

Tregs’ ability to modulate immune responses and infiltrate cutaneous tissues strongly implicates them in AD pathogenesis (216). Genetic conditions that disrupt Treg function—including Immunodeficiency, Polyendocrinopathy, Enteropathy, X-linked (IPEX) syndrome and Wiskott-Aldrich syndrome (WAS)—offer strong support for this involvement (217). These disorders involve Treg dysfunction due to FoxP3 mutations or defective Wiskott-Aldrich syndrome protein, respectively, and their associated eczematous skin lesions closely resemble AD, underscoring the role of impaired Tregs in AD development (218). Similarly, Treg-deficient scurfy mice develop eczematous dermatitis that mimics AD lesions. Additionally, several treatments for allergic disorders that generate or modulate Treg function—such as allergen immunotherapy (AIT) and vitamin D supplementation—have demonstrated clinical benefits in AD patients, potentially by increasing Treg numbers or enhancing their suppressive capacity (219, 220).

In the context of AD-related immune dysregulation, the role of the altered cutaneous microbiome in driving Treg dysfunction is particularly noteworthy. Skin Tregs colocalize with commensal bacteria at hair follicles (221, 222), and their reduced abundance in germ-free mice highlights the microbiome’s role in promoting skin Treg expansion (223). The cutaneous microbiome of AD patients is typically dominated by *Staphylococcus aureus* (*S*. aureus) strains, many of which secrete superantigenic toxins (223). Patients with severe AD often exhibit a predominance of S. aureus, whereas *Staphylococcus epidermidis* is more prevalent in milder disease (224). Notably, *S. aureus* isolates from AD patients experiencing severe flares induce epidermal thickening and expansion of cutaneous Th2 and Th17 cells in a murine skin colonization model (225). A related study found that impaired skin Treg-mediated immunoregulation promotes type 2 cytokine production by commensal-specific plastic Th17 cells (226). These findings point to a convergence of cutaneous processes in AD: skin Treg dysfunction, dysregulated Th2 immunity, and microbiome alterations—all of which merit further investigation.

In summary, Tregs’ contribution to AD pathogenesis is multifaceted and continues to be an area of active investigation. Defects in Treg function and their interactions with the cutaneous microbiome are clearly implicated in disease development. A comprehensive phenotypic and mechanistic analysis of Tregs during AD cutaneous flares is essential to better understand their role in the disease and to guide the development of effective Treg-based therapeutics for AD.

### 4.3 Regulatory T cells as central mediators of tolerance in autoimmune and allergic diseases

Conventionally, autoimmune diseases—often characterized by Th1/Th17-predominant responses—and allergic disorders—primarily driven by Th2 signaling cascades—have been viewed as distinct immunopathological conditions. Yet, through the prism of regulatory T cells (Tregs), both disease categories unite around a common fundamental impairment: the breakdown of core immune tolerance mechanisms. This shared feature is illustrated in **Table 2**, which outlines aberrant Treg alterations across both autoimmune and allergic spectra. A defining trait of both disease types lies in Treg abnormalities, either numerical reductions or functional deficits. For instance, in autoimmune conditions like RA and SLE, Treg populations may show diminished counts or compromised suppressive capacity (86). Similarly, in allergic disorders such as AD and asthma, Treg numbers might remain unaltered or even elevated, but their suppressive efficacy is frequently impaired—especially in the context of allergen-specific immune responses (142, 146). Beyond this overlap in Treg function, both disease groups exhibit disruptions in the cytokine microenvironment and Treg/Th17 balance. In autoimmune settings like psoriasis, as well as allergic manifestations such as severe AD, inadequate Treg-mediated suppression coincides with heightened Th17 cell activity (135, 136). While transforming growth factor-β (TGF-β) acts as a key driver of Treg differentiation, its crosstalk with proinflammatory mediators like interleukin-6 (IL-6) can redirect cellular differentiation toward Th17 lineages. These Th17 cells then secrete cytokines such as IL-17, perpetuating inflammatory tissue damage. As a result, the inflammatory microenvironment in both autoimmune and allergic diseases contributes to pathogenesis by disrupting the Treg/Th17 equilibrium. Furthermore, Treg function and differentiation are profoundly shaped by epigenetic regulation and microbial cues. Gut commensal bacteria—including Clostridium species—facilitate Treg maturation and functional competence, with their metabolites (e.g., short-chain fatty acids, SCFAs) enhancing Treg performance through mechanisms such as histone deacetylase (HDAC) inhibition (188–190). Thus, microbiota dysbiosis emerges as a key contributing element to the development of both disease categories.

**Table 2:** Abnormal changes of Treg cells in autoimmune and allergic diseases.

Disease type	Representative disease	Changes in Treg number	Abnormal Treg function	Key mechanisms	References
Autoimmune Diseases	Rheumatoid Arthritis	Normal/Increased	Functional Suppression	Inflammatory Factors (TNF-α, IL-6) Suppressive Function	(78)
	Systemic Lupus Erythematosus	Reduced	Unstable Foxp3 Expression	Inadequate IL-2 Production; Epigenetic Alterations	(86)
	Multiple Sclerosis	Normal	Migration Defects	Abnormal Chemokine Receptor Expression	(93)
	Type 1 Diabetes	Reduced	Impaired Suppressive Function	Reduced IL-2 Signaling; Teffs Resist Suppression	(110)
Allergic Diseases	Allergic Rhinitis	Normal/Reduced	Decreased Function During Seasonal Disease Activity;	Allergen Exposure Affects Function	(142, 146)
	Asthma	Normal	Impaired Suppressive Function	Reduced Pulmonary Treg Stability	(166)
	Food Allergy	Reduced	Antigen-Specific Tregs Inadequate	IL-10 Production	(194)
	Atopic Dermatitis	Normal/Increased	Defective Skin Barrier Repair	Disruption of TSLP/IL-33 Signaling	(214)

Given these convergent immunoregulatory pathways, Treg-targeted therapeutic modalities hold wide relevance across autoimmune and allergic disorders. These interventions aim to reinstate Treg-orchestrated tolerance and rectify underlying immune imbalances, moving beyond mere symptomatic control of inflammation to address root pathogenic mechanisms—representing a vital direction for future treatments. Key therapeutic strategies include: firstly, low-dose IL-2 administration to enhance *in vivo* Treg expansion and functionality; secondly, adoptive Treg transfer following ex vivo expansion of autologous or allogeneic cells; thirdly, antigen-specific immunotherapy to induce dedicated Treg populations via peptide-based or allergen desensitization approaches (219, 220); fourthly, epigenetic modulation using HDAC inhibitors or histone acetyltransferase (HAT) regulators to strengthen Treg competence (62); and lastly, microbiome-directed interventions such as probiotics or prebiotics to modulate Treg differentiation (197).

## 5 Therapeutic approaches of Tregs-based immunotherapy

### 5.1 Immunosuppressive agents

#### 5.1.1 Corticosteroids

**Figure 5:**
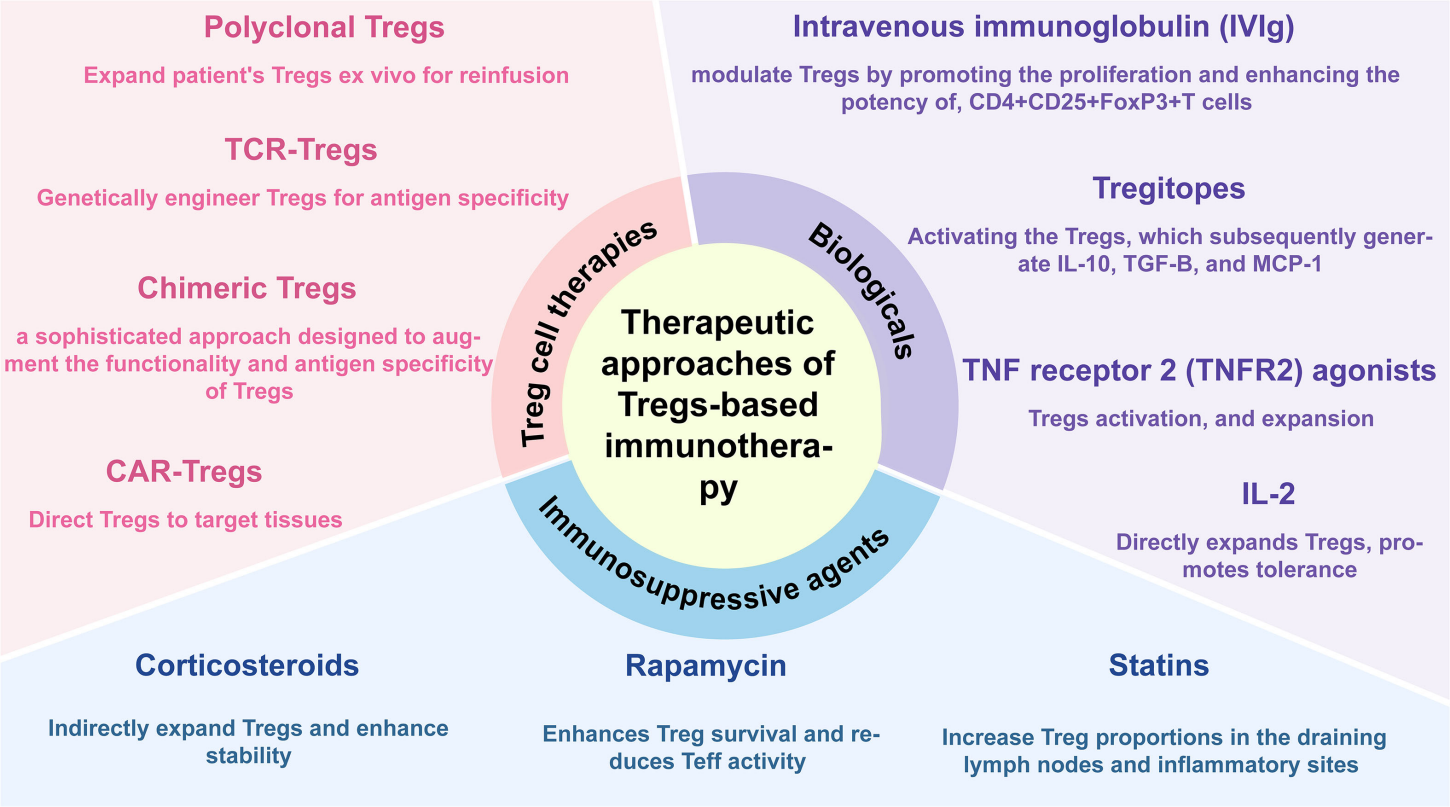
Therapeutic approaches of Tregs-based immunotherapy. This figure summarizes current and emerging strategies that target or harness regulatory T cells (Tregs) to restore immune tolerance in autoimmune and allergic diseases. These approaches fall into three main categories: Conventional Immunosuppressants- Glucocorticoids, Rapamycin and statins; Biologicals- Interleukin-2 (IL-2), TNF receptor 2 (TNFR2) agonists, Intravenous immunoglobulin (IVIg) and Tregitopes; Treg-based Therapies- Polyclonal Tregs therapy, Engineered antigen-specific TCR-Treg therapy, Chimeric Treg therapy and Chimeric antigen receptor (CAR)-Tregs therapy. These complementary approaches modulate Tregs to reprogram immune homeostasis and advance precision immunotherapy for autoimmune and allergic diseases.

Corticosteroids represent extensively utilized immunosuppressive compounds, notably in managing severe allergic responses, autoimmune disease exacerbations, and post-organ transplantation care (227) (see **Figure 5**). Corticosteroids -mediated therapeutic benefits typically endure for several days to weeks post-administration, stemming from their extensive influence on the development and differentiation of diverse immune cell populations—including T cells, DCs, and macrophages (228–230).

Corticosteroids exert a profound impact on T cell viability, maturation, and lineage commitment. They trigger apoptosis in conventional T cells (Tconv), consequently elevating the Treg/Tconv ratio. Beyond this, Corticosteroids enhance Treg abundance and functional potency through both direct and indirect cellular pathways (231). Directly, they stimulate TGF-β receptors, which precipitates the phosphorylation and nuclear translocation of SMAD2 and SMAD3 proteins. These signaling molecules then bind to the FoxP3 promoter, boosting FoxP3 expression and driving Treg differentiation from naive T cells (232). Indirectly, Corticosteroids modulate non-T cell populations like plasmacytoid DCs, which foster Treg development via both TLR-dependent and TLR-independent mechanisms (232). Additionally, they promote Treg expansion by inducing non-T cells to secrete increased levels of TGF-β (231).

In autoimmune diseases, Corticosteroids have been demonstrated to augment Treg proportions in a dose-responsive fashion—an effect observed in patients with SLE (233). High-dose dexamethasone therapy in individuals with immune thrombocytopenic purpura (ITP) also elevated Treg counts, with CD25^+^CD127^-^ Tregs reaching peak levels 14 days after Corticosteroids administration (234). In allergic disorders, studies focusing on asthma have shown that Corticosteroids can enhance Treg numbers and functionality. For example, topical Corticosteroids administration upregulated FoxP3 mRNA expression in peripheral blood mononuclear cells (PBMCs) of patients with moderate asthma (235). This was associated with elevated concentrations of IL-10 and TGF-β, suggesting these cytokines either originate from Tregs or promote the differentiation of pTregs. Another study reported that children with asthma receiving inhaled corticosteroids exhibited higher percentages of CD4^+^CD25^high^ T cells in their PBMCs and bronchoalveolar lavage fluid (BALF), with Corticosteroids restoring the suppressive activity of these Tregs (165). Similarly, a murine model of ovalbumin-induced asthma exhibited expanded Treg populations following prolonged Corticosteroids exposure, supporting the notion that mid- to long-term Corticosteroids effects include Treg expansion alongside immediate anti-inflammatory actions (236). Collectively, Corticosteroids administration in asthmatic patients yields both sustained increases in Treg counts and anti-inflammatory benefits.

Notably, Corticosteroids exert disease- and tissue-specific impacts on Treg numbers. While Corticosteroids-treated patients with SLE, ITP, asthma, and nickel allergy display increased Treg counts, the converse has been documented in psoriasis patients (237). Additionally, the outcomes of high-dose Corticosteroids in relapsed MS patients remain inconclusive (237). In summary, the influence of Corticosteroids treatment on Treg counts varies by disease type and tissue involvement. Given that Treg expansion may be pivotal for disease control, Corticosteroids treatment planning necessitates consideration of their effects on Treg counts—an aspect critical for optimizing therapeutic outcomes in immune-mediated disorders.

#### 5.1.2 Rapamycin

Rapamycin (sirolimus), a macrolide antimicrobial compound derived from *Streptomyces hygroscopicus*, serves as a potent agent for mitigating allograft rejection (238) (see **Figure 5**). Like cyclosporine A and FK506 (tacrolimus), it interacts with the intracellular immunophilin FK506-binding protein (FKBP12) (239). Unlike these immunosuppressants—which obstruct T-cell receptor (TCR)-triggered activation—rapamycin dampens cytokine-mediated signaling through its action on the mammalian target of rapamycin (mTOR), a serine/threonine kinase indispensable for protein biosynthesis and cell cycle advancement. Studies have demonstrated that rapamycin selectively fosters Treg expansion, and these cells exert robust protection against allograft rejection by suppressing the proliferation of syngeneic T cells in both *in vitro* and *in vivo* settings (240). Furthermore, rapamycin emerges as a viable candidate for ex vivo Treg expansion in T cell-driven pathologies, as it fails to hinder activation-induced cell death or CD4+ T cell proliferation under *in vitro* conditions (241). Rapamycin’s dual capacity to diminish T effector (Teff) cells while facilitating Treg differentiation renders it a valuable tool for developing innovative and safe cellular immunotherapeutic strategies (242).

#### 5.1.3 Vasoactive intestinal peptide and statins

Vasoactive intestinal peptide (VIP) has been demonstrated to enhance Treg differentiation and proliferation within peripheral tissues and joints alike. VIP-induced Tregs have been shown to exert substantial suppressive effects and ameliorate chronic autoimmune conditions, establishing VIP as a promising therapeutic candidate for Treg-centric immunotherapy (243) (see **Figure 5**). Statins—commonly prescribed for their cardioprotective advantages—additionally exhibit pleiotropic immunomodulatory and anti-inflammatory activities that extend beyond their cholesterol-lowering effects. Their ability to selectively elevate Treg proportions in inflammatory foci and draining lymph nodes underscores their potential as Treg-targeted therapies for immune-mediated disorders (244, 245).

### 5.2 Biologicals

#### 5.2.1 Interleukin-2

Interleukin-2 (IL-2) is a crucial proinflammatory cytokine that plays a major role in immune regulation and microbial defense, primarily through its effects on Tregs (246) (see **Figure 5**). The importance of IL-2 in Treg development and immune homeostasis is highlighted by the observation that IL-2-deficient mice exhibit uncontrolled T cell activation and autoimmunity (247). The IL-2 receptor (IL-2R), which can be either a high-affinity heterotrimeric complex (IL-2R α/CD25, IL-2Rβ, and γc chains) or a heterodimer (IL-2Rβ/CD122 and common γ (γc)/CD132 chains), is where IL-2 binds to produce its effects (248). Tregs constitutively express IL-2R α at high levels, making them more responsive to IL-2 signaling than other immune cells, including NK, Tconv, and innate lymphoid cells, which also express CD25, but to a lesser extent (249). This is because they constitutively express IL-2R α at high levels. IL-2 activates the signaling pathways that control Treg homeostasis and function, including STAT5/JAK1/3 and, to a lesser extent, PI3K/AKT/mTOR (250). IL-2 signaling suppresses the expression of immune checkpoint molecules on Tregs, including CTLA-4 and PD-1, while promoting Treg proliferation (251). Considering these characteristics, reestablishing immunological tolerance and preventing pathogenic autoimmune reactions can be achieved by focusing on the IL-2/Treg axis.

The FDA approved high-dose IL-2 therapy for metastatic cancers in 1992, but it’s extremely short half-life (less than 10 minutes) necessitated a high-dose bolus, which resulted in severe side effects like cytokine storm and vascular leak syndrome (252). Low-dose IL-2 (ld-IL-2) selectively expands Tregs with minimal activation of effector T cells; a multicenter trial demonstrated its broad immunoregulatory efficacy and safety across autoimmune diseases (253). In RA, ld-IL-2 combined with methotrexate increased circulating Tregs, improved immunological profiles, and alleviated clinical symptoms in a phase II study (254). Recent research has also shown that IL-2-based therapeutic approaches can effectively induce tolerance in animal models of food allergy and allergic asthma (255, 256). It is hypothesized that the selective expansion of Tregs via IL-2 administration may be beneficial for treating food allergies. The safe and well-tolerated nature of low-dose IL-2 has been confirmed in preclinical and clinical studies, paving the way for next-generation IL-2-based treatments (257).

#### 5.2.2 TNF receptor 2 agonists

TNF-α, a pro-inflammatory cytokine, binds to two distinct receptors: TNFR1, which has a pro-inflammatory effect, and TNFR2, which promotes tissue regeneration and reduces inflammation (258) (see **Figure 5**). Tregs have been shown to express more TNFR2 than other T cell subsets, and their suppressive activity is directly correlated with TNFR2 expression (259). The molecular mechanisms underlying TNF-TNFR2 signaling in Tregs are complex and involve several key pathways. This signaling promotes the preferential expansion of Tregs by suppressing DNA methylation at the *FoxP3* promoter (260). It also maintains an autocrine TNF-TNFR2 feedback loop that ensures Treg stability (258). Furthermore, it regulates kinase activities linked to TCR, JAK, MAPK, and PKC signaling pathways and modulates IL-17 expression in human Tregs (261). By promoting Treg function and proliferation, TNFR2 agonists may offer a novel therapeutic approach for treating various inflammatory and autoimmune conditions (262).

#### 5.2.3 Intravenous immunoglobulin

Intravenous immunoglobulin (IVIg) is a sterile therapeutic preparation containing human IgG derived from a pool of healthy plasma donors (263) (see **Figure 5**). Administered at high doses (1 to 3 g/kg), IVIg has proven effective in treating a range of neurological and autoimmune conditions, including immunothrombocytopenia, Kawasaki disease, Guillain-Barré syndrome, chronic inflammatory demyelinating polyneuropathy, SLE, and dermatomyositis (264). IVIg infusions have been shown to modulate Tregs by enhancing the proliferation and potency of CD4^+^CD25^+^FoxP3^+^ T cells. This effect was demonstrated in NK cell-dependent EAE models. Following IVIg administration, patients with autoimmune rheumatic diseases also showed Treg expansion, which was linked to an increase in DCs cyclooxygenase-2-dependent prostaglandin E2 synthesis (265).

#### 5.2.4 Tregitopes

Tregitopes are a novel class of peptides (15–26 amino acids in length) crucial for maintaining immune system control. They work by activating Tregs, which in turn produce suppressive cytokines like IL-10, TGF-β, and MCP-1 (266) (see **Figure 5**). This discovery has opened up the possibility of using Tregitopes as therapeutic targets for autoimmune diseases, including MS, SLE, and T1D (267). The primary mechanism by which Tregitopes induce immune tolerance is through their binding to MHC II molecules on the surface of APCs, which allows them to be presented to and activate Tregs (267). Furthermore, Tregitopes suppress the NF-κB pathway, which reduces the expression of costimulatory molecules and increases T cell anergy. They also decrease the expression of T effector (Teff) cytokines (IFN-γ, IL-5, and IL-6) while simultaneously increasing the production of Treg-associated cytokines (IL-10 and TGF-β). Tregitopes can also promote the conversion of Th2 cells into adaptive Tregs and Th1-like phenotypes by upregulating CTLA-4 expression (266).

Computational tools are used to identify Tregitopes within IgG, classifying peptides based on factors like their binding strength with MHC-II (267). Tregitopes have recently been used to treat autoimmune diseases primarily through Treg activation (268). For example, studies in T1D have explored using insulin-derived peptides to restore Treg activity. The C19-A3 peptide showed promise in a Phase I clinical trial, preserving islet cell function, whereas the B9–23 peptide resulted in anaphylactic reactions due to its unpredictable interaction with MHC (269). A safer approach for treating MS involved the co-administration of IVIg-derived Tregitopes with the MOG35–55 epitope, as this combination showed significant anti-inflammatory properties in EAE models (266). More recently, Bemani et al. (270) used bioinformatics to create a multi-epitope vaccine to increase tolerance in myelin-specific T cells, which could slow MS progression and prevent relapses. This vaccine includes an anti-DEC205 single-chain variable fragment (scFv) antibody, a multi-epitope section with MS-associated antigens and Tregitopes, and VIP. Additionally, Edratide, a human peptide not classified as a Tregitope but with a similar function, has been shown to reduce SLE symptoms by modulating TGF-β, FoxP3, and inflammatory cytokines like IFN-γ and IL-1β, thereby restoring immune homeostasis (271, 272). Despite their promise, Tregitope-based therapies face several challenges, including the need to optimize drug formulations and delivery methods to specifically target Tregs. More preclinical and clinical research is required to determine the best dosages and delivery systems (266). In a study on a mouse model of allergic airway disease (AAD), Marieme Dembele et al. (273) found that administering mouse and human IgG Tregitopes decreased lung inflammation and attenuated allergen-induced airway hyperresponsiveness. Similar to IVIg, human IgG Tregitopes reduced allergic airway disease in mice. Tregitope treatment increased Helios^+^ Tregs in mediastinal lymph nodes, and Tregs from treated mice showed enhanced suppression compared to controls. The antigen-specific nature of the Treg response was confirmed when transferring Tregs from treated mice to allergen-sensitized mice; only Tregs from mice exposed to the same allergen were able to reduce AAD (273).

In conclusion, the combination of Tregitopes and allergens offers a natural immune tolerance mechanism that may induce highly suppressive and antigen-specific Helios^+^ Tregs. This form of immunomodulation holds promise as a new treatment for human reactive airway disease and other allergic disorders.

### 5.3 Treg cell therapies

#### 5.3.1 Polyclonal Tregs therapy

Treg cell therapy aims to restore immune tolerance by directly increasing the number of Tregs, especially in autoimmune disorders where autoantigen-specific Treg function is compromised (274) (see **Figure 5**). The effectiveness of this strategy relies on the ability of Tregs to maintain their suppressive function and stability over time while retaining antigen specificity (275).

In polyclonal Treg therapy, CD4^+^CD25^high^ Tregs are isolated from the patient’s peripheral blood or inflammatory sites, expanded *in vitro*, and then re-infused. When stimulated with anti-CD3/CD28 monoclonal antibody-coated beads and high doses of IL-2, human Tregs can proliferate up to 40,000-fold *in vitro*. These expanded cells exhibit stronger suppressive function than freshly isolated Tregs while maintaining their expression of CD25, FoxP3, and lymph node homing receptors (276). However, the expansion and infusion of Tregs present several challenges: (i) the protocol is labor-intensive for each patient; (ii) it requires careful monitoring to ensure the purity and antigen specificity of the Tregs to prevent the proliferation of self-reactive Teff cells or Tregs with incorrect specificity; and (iii) there is a potential risk of infection or transformation during the *ex vivo* expansion process (277). After infusion, the injected Tregs peak in the bloodstream within the first two weeks, then gradually decline, but can remain detectable for up to a year (278).

Polyclonal Treg therapy is currently being investigated in clinical trials for conditions like T1D, COVID-19, cutaneous pemphigus, autoimmune hepatitis (AIH), and SLE aming to optimize treatment by determining the ideal dosage, number of doses, and timing between infusions (279). Better therapeutic outcomes and enhanced Treg suppressive capacity are expected when Treg therapy is combined with other treatments that increase Treg numbers and function, such as rapamycin, TNFR2 agonists, IL-2, and IL-10 (275). There is still great potential in this field, particularly in developing methods to improve Treg resistance and persistence for more durable and effective immunomodulation.

#### 5.3.2 Engineered antigen-specific TCR-Treg therapy

Incorporating an autoantigen-specific T-cell receptor (TCR) can enhance Treg therapy by focusing the cells’ response on a particular autoantigen, thereby avoiding the risk of widespread immune suppression (280) (see **Figure 5**). This approach involves genetically modifying Tregs *ex vivo* using retroviral or lentiviral transduction methods to express a high-affinity, autoantigen-specific TCR. These modified TCR-Tregs can then be expanded and re-infused into patients to regulate disease-specific autoimmune responses (281).

Recent studies have demonstrated that combining TCR knock-in with FOXP3 stabilization or engineered IL-2–responsive modules markedly improve Treg stability, persistence, and antigen-dependent activation. A representative example is GNTI-122, a dual-edited islet-specific TCR-Treg product incorporating FOXP3 reinforcement and a chemically inducible IL-2 signaling cassette, which showed robust antigen-specific activation, pancreatic homing, and suppression of diabetogenic responses in preclinical models of type 1 diabetes (T1D) (282).

Antigen-specific TCR-Tregs have been most actively developed for organ-specific autoimmune diseases with well-defined autoantigens. In multiple sclerosis (MS), myelin basic protein (MBP) and myelin oligodendrocyte glycoprotein (MOG) remain principal targets, and engineered myelin-specific TCR-Tregs suppress pathogenic effector T-cell responses and ameliorate disease in experimental autoimmune encephalomyelitis models (277, 283). In T1D, insulin, glutamic acid decarboxylase (GAD), IGRP, and hybrid insulin peptides have been used to generate islet-reactive TCR-Tregs capable of controlling autoreactive T-cell expansion and limiting insulitis (277, 282). These advances underscore critical translational considerations, including stringent TCR specificity screening, HLA restriction, and incorporation of persistence/survival modules to ensure durable functionality in inflammatory microenvironments (283).

Beyond autoimmunity, antigen-specific regulatory strategies are rapidly emerging in allergic diseases, in which allergen-specific tolerance is central to long-term disease control. Allergen-specific immunotherapy (AIT) naturally induces allergen-directed FOXP3+ Tregs, and engineered TCR-Tregs are now being explored to mimic and enhance this pathway. Preclinical studies suggest that allergen-specific regulatory cells can suppress IgE production, Th2 cytokines, and effector-cell activation in models of allergic rhinitis and asthma, although challenges remain regarding allergen heterogeneity, mucosal trafficking, and long-term Treg stability (284). Contemporary reviews highlight that integrating antigen specificity with engineered stability modules represents a promising avenue to achieve durable immune tolerance across both autoimmune and allergic contexts (275, 285).

#### 5.3.3 Chimeric Treg therapy

Chimeric Treg therapy is an advanced technique designed to enhance the antigen specificity and functionality of Tregs. This strategy involves the genetic modification of Tregs to express specific proteins on their surface, such as MHC class II-restricted TCRs (286) (see **Figure 5**). In theory, any desired protein can be transduced onto a Treg cell. Numerous innovatively designed chimeric Tregs have already shown their efficacy in preclinical models of autoimmune diseases. In one MS mouse model, for example, the cytoplasmic tail of CD3ζ was associated with a myelin peptide-MHC class II complex. Treg effectiveness was at least ten times increased as a result. The enhancement was further regulated by co-transduction of FoxP3 and the peptide-MHC class II complex onto Tconv cells (287). Furthermore, it has been demonstrated that Tregs retained their antigen-specific suppressive activity when MHC class I-restricted TCRs were transduced onto them (288). This implies that TCRs from non-Treg cells, like CD8^+^ CD4^+^ T cells or Tconv cells, could be successfully transferred onto Tregs in order to reroute their regulatory function (289).

#### 5.3.4 Chimeric antigen receptor-Tregs therapy

Chimeric Antigen Receptor (CAR)-Tregs are a specialized type of chimeric Treg designed to directly target specific tissue autoantigens without relying on MHC restriction. The CAR molecule consists of an extracellular antigen-recognition domain, a hinge, a transmembrane region, and an intracellular signaling domain that is part of the Treg signaling machinery (290) (see **Figure 5**). CAR-Tregs are engineered to migrate to and attach to tissue-specific autoantigens at the site of autoimmunity, thereby concentrating their suppressive effects (291). However, a potential drawback is that if the target autoantigen is present in healthy tissues, widespread activation of CAR-Tregs could occur, leading to undesirable systemic immune suppression (**Table 3**).

**Table 3:** Human studies of CAR-T cell treatment for autoimmune diseases.

Disease	Target Ag	CAR T cell/Treg	Study title/Study summary	Current status	Clinical trial
MPV	Dsg3	Dsg3 CAAR-T cells (CD137-CD3ζ)	Safety and dosing study of autologous DSG3-CAART in subjects with active MPV. being conducted	Phase I recruiting	NCT04422912
NMOSD	CD19/CD20	Anti-CD19/20-CAAR-T (tanCART19/20)	The purpose of this study was to assess the safety and efficacy of this tanCART19/20 in the treatment of NMOSD. Withdrawn (It was hard to recruit patients)	Phase I Withdrawn	NCT03605238
NMOSD	BCMA	anti-BCMA CAAR-T	The safety and efficacy of a novel CAR-T cell therapy using CT103A cells, are evaluated in patients with relapsed/refractory NMOSD. being conducted	Phase I recruiting	NCT04561557
Lupus SLE	CD19	CD19-targeted CAAR-CD8 + T (CD28-CD3ζ)	assess the safety and efficacy of this CAR-T cells in the treatment of SLE. Their effects still yet to be known	Phase I	NCT03030976
MG	BCMA	anti-BCMA CAR-T (Descartes-08 CAR-T cells)	Descartes-08: the half-life of is well-characterized. No uncontrolled reproduction. No severe toxicity	In progress/not accepting new patients	NCT04146051
SLE, SS	CD19 (anti-B cell)	Anti-CD19 CAR-T cells (autologous)	Study to assess safety of anti-CD19 CAR-T in subjects with active, B-driven autoimmune disease (SLE, SSc, DM/PM) — safety/tolerability endpoints.	Phase 1 Phase 2	NCT06347718
HLA-A2 mismatched liver transplant	HLA-A2 (alloantigen)	Autologous antigen-specific CAR-Treg (QEL-001; HLA-A2 CAR-Treg)	First-in-human LIBERATE study: autologous HLA-A2-specific CAR-Treg (QEL-001) to promote donor-specific tolerance after HLA-A2 mismatched liver transplant.	Phase I recruiting	NCT05234190.
MuSK-MG	MuSK — CAAR	Autologous MuSK-CAART	Phase I open-label safety study to evaluate various dosing regimens of MuSK-CAART to selectively deplete anti-MuSK B cells.	Phase I recruiting	NCT05451212

In contrast to TCR-Tregs, CAR-Tregs have a higher affinity for their corresponding antigen, circumvent MHC restriction, and are less reliant on IL-2. These characteristics set CAR technologies apart from TCR engineering. For them to effectively stimulate Tregs, the target cell must have at least 100 target autoantigens. By contrast, the TCR can activate the Treg population with just one peptide-MHC complex (92). Since TCRs are normally detected at a level of ~50,000/cell, while CARs are present in greater quantities, surpassing 50,000/cell, this difference should be taken into account. Immunoreceptor tyrosine-based activation motifs (ITAMs) and tyrosines are more abundant in the intracellular signaling domain of CAR-Tregs than in TCR-Tregs, which results in a more potent activation and a greater signaling capacity. TCRs are easier to express because of their heterodimeric structure, whereas CAR is a monomeric protein. Co-stimulatory receptors like CD4 and CD28 are necessary for TCR activation. Nevertheless, CAR does not require these coreceptors for activation (292).

The CAR-Treg approach has shown promise in preclinical models. Recently, a mouse model of ovalbumin (OVA) allergy was used to assess the CAR approach. The CAR Treg cell in this study was made up of OVA connected to the CD28-CD3ζ transmembrane and signal transduction domains (293). In the mice model, this CAR Treg cell therapy reduced the anaphylactic reaction brought on by intraperitoneal OVA injection (293). Treg cells transduced with Bet v 1-specific TCR inhibited the production of cytokines and the proliferation of allergen-specific effector T cells in cell culture (294).

The use of allogeneic CAR-T cells from “healthy donors” offers a rapid, scalable, and cost-effective method for producing large quantities of cells that can be cryopreserved and made readily available for patients. However, a major challenge is that the host immune system may reject them, or they could cause graft-versus-host disease (GvHD) (295). Consequently, novel approaches are being explored to generate “off-the-shelf” universal CAR-T cells from healthy allogeneic donors. One such approach involves gene editing using platforms like clustered regularly interspaced short palindromic repeats (CRISPR)/Cas9 (296).

## 6 Challenges in the clinical translation of Treg-based therapies for allergic rhinitis and autoimmune diseases

Despite strong evidence from how they work and promising early lab results, the use of Treg-based treatments for allergic rhinitis (AR) and autoimmune diseases in clinics is held back by several unsolved problems. These challenges cover Treg production, stability, tissue targeting, safety, clinical plan design, lab model limits, and large-scale production.

Getting enough pure FoxP3^+^CD25^high^ Tregs for treatment is a major hurdle. Therapy usually needs 10^6^–10^8^ cells per kilogram of body weight, but growing Tregs outside the body often leads to mixing with harmful effector T cells. Strict purification reduces the number of Tregs obtained—this is even worse for autoimmune patients, who often start with too few Tregs. Another problem is functional instability: inflamed environments rich in certain proteins (IL-4/IL-13 in AR and IL-6/TNF-α in autoimmune diseases) can lower FoxP3 levels and turn Tregs into effector-like or even harmful cells. While gene-editing and protein-based treatments can improve stability, concerns about unintended effects, different patient responses, and long-term effectiveness remain.

Beyond production challenges, targeted delivery and tissue homing pose significant troubles. Giving Tregs through the veins makes most of them get stuck in non-target organs like the liver and spleen, so too few reach the diseased areas (e.g., nasal lining in AR or joints/kidneys in autoimmune diseases). Local delivery also has issues: mucus barriers and damaged nasal lining in AR stop Tregs from staying alive, while inflamed blood vessels and changed protein signals in autoimmune tissues block Tregs from entering. The lack of good delivery tools—such as tissue-targeting materials, protein-guided carriers, or tiny particle delivery systems—further limits precise homing.

Safety remains a critical concern for Treg therapy. It has inherent risks of too much immune suppression, which may increase infection risk or weaken the nasal lining’s defense against AR. Other safety issues include genetic changes when growing cells outside the body, the risk that donated Tregs trigger an immune response, and unknown long-term effects like abnormal immune tolerance or shifts in overall immune function. Importantly, most current clinical trials don’t follow patients for long, so we don’t fully understand how long transferred Tregs last, how well they work over time, or their long-term safety.

Clinical protocol design also presents significant uncertainties. Key clinical details—including the best dose, delivery method, treatment frequency, and length—are not well defined. Effective doses seem to differ by disease and disease stage, and the range between not working and suppressing the immune system too much is narrow. Local nasal delivery for AR (e.g., nebulized or nose drops) is still experimental. Furthermore, combining Treg therapy with other treatments—such as steroids, JAK inhibitors, allergen immunotherapy, or antigen-specific tolerance induction—has not been fully optimized or standardized.

Compounding these issues are gaps in preclinical modeling and biomarker development. Common mouse models (e.g., OVA-induced AR or collagen-induced arthritis) don’t fully reflect human immune differences, Treg stability, or chronic disease progression. As a result, lab results often don’t predict how well treatments work in humans. The lack of reliable markers to select patients, monitor Treg function in real time, and spot early safety issues also makes it hard to standardize clinical outcomes and accurately assess treatment effects.

Finally, technical and industrial barriers hinder widespread translation. Producing large amounts of clinical-grade Tregs is technically difficult. Growing protocols rely on complex protein mixes and stimulation systems that vary between labs, making standardization and quality control hard. Costs for making patient-specific Tregs are very high, while using donated Tregs raises issues of immune compatibility and unknown long-term survival. Together, these technical and economic barriers limit wider clinical use.

## 7 Conclusion

Tregs are pivotal in sustaining immune homeostasis and peripheral tolerance. Deviations in their abundance or functionality are intimately linked to the pathogenesis of autoimmune and allergic diseases. Although these disorders present with disparate clinical features, they converge on a fundamental defect in Treg-orchestrated tolerance mechanisms, positioning Tregs as a crucial nexus between these principal immunological categories. Over recent years, diverse therapeutic modalities have demonstrated encouraging efficacy in reinstating immune equilibrium, as evidenced by preclinical models and clinical investigations. These progressions indicate that precision Treg-directed therapies are evolving into feasible clinical options. Nevertheless, Treg-based interventions encounter formidable challenges, including the intrinsic heterogeneity and plasticity of Tregs, the erosive effects of inflammatory microenvironments, and the risk of Treg transdifferentiation into proinflammatory states amid chronic inflammation. Prospective inquiries should prioritize delineating the molecular underpinnings of Foxp3 stability and Treg adaptability to enable the formulation of tissue- and antigen-tailored precision strategies. Integrating surface biomarker profiling, multi-omics surveillance, and translational methodologies offers substantial promise for realizing individualized immune restoration and enduring tolerance. In essence, Tregs not only constitute a central pathological fulcrum in autoimmune and allergic afflictions but also embody a prime avenue for attaining meticulous immune recalibration and sustained tolerance prospectively.

## Glossary

AAD: allergic airway disease
AD: atopic dermatitis
AHR: aryl hydrocarbon receptor
AIG: autoimmune gastritis
AIH: autoimmune hepatitis
AIT: allergen immunotherapy
APCs: antigen-presenting cells AR, allergic rhinitis
Areg: amphiregulin
BALF: bronchoalveolar lavage fluid
Bcl-2: B-cell lymphoma 2
BCMA: B cell maturation antigen
CAART: chimeric antigen receptor-T cell therapy
CAR: chimeric antigen receptor
CB4: Coxsackievirus B4
CCR: C-C Chemokine Receptor
CD: cluster of differentiation
CKD: chronic kidney disease
CNS: central nervous system
CO: carbon monoxide
COVID-19: corona virus disease 2019
CpG: cytosine-phosphate-Guanine
CRISPR: clustered regularly interspaced short palindromic repeats
CRTH2: chemoattractant receptor-homologous molecule expressed on Th2 cells
CTLA-4: cytotoxic T-lymphocyte-associated protein 4
DCs: dendritic cells
DNA: deoxyribonucleic acid
Dsg3: desmoglein 3
EAE: experimental autoimmune encephalomyelitis
EAT: experimental autoimmune thyroiditis
FA: food allergy
FcεRI: Fc epsilon receptor I
FDA: food and drug administration
FHA: filamentous hemagglutinin
FKBP12: FK506-binding protein
FoxP3: forkhead box P3
GATA3: GATA binding protein 3
GBM: glomerular basement membrane
GFP: green fluorescent protein
GM-CSF: granulocyte-macrophage colony-stimulating factor
GvHD: graft-versus-host disease
HDAC: Histone Deacetylase
HT: Hashimoto’s thyroiditis
IBD: inflammatory bowel disease
IDO: indoleamine 2,3-dioxygenase
IFN: interferon
IgE: immunoglobulin E
IgG: Immunoglobulin G
IL: interleukin
ILCs: innate lymphoid cells
ILT3^+^: immunoglobulin-like transcript 3-positive
IPEX: immunodeficiency, polyendocrinopathy, and enteropathy X-linked syndrome
ITAMs: immunoreceptor tyrosine-based activation motifs
ITP: immune thrombocytopenic purpura
iTr35: inducible regulatory T cells 35
iTregs: induced Tregs
IVIg: Intravenous immunoglobulin
JAG1: Jagged 1
JAK: Janus kinase
JIA: juvenile idiopathic arthritis
MAPK: mitogen-activated protein kinase
MBP: myelin basic protein
MHC: major histocompatibility complex
MK: midkine
MLN: mesenteric lymph nodes
MOG: myelin oligodendrocyte glycoprotein
MOG: myelin oligodendrocyte glycoprotein
MPV: metapneumovirus
MS: multiple sclerosis
mTOR: mammalian target of rapamycin
NFAT1: nuclear factor of activated T cells 1
NF-κB: nuclear factor-kappa B
NKs: natural killer cells
NMOSD: neuromyelitis optica spectrum disorder
NO2: nitrogen dioxide
NOD: non-obese diabetic
nTregs: natural Tregs
OVA: ovalbumin
PAHs: polycyclic aromatic hydrocarbons
PBMCs: peripheral blood mononuclear cells
PD: parkinson’s disease
PD1: programmed death-1
pDCs: plasmacytoid dendritic cells
PKC: protein kinase C
PM: particulate matter
PPAR-γ: peroxisome proliferator-activated receptor-gamma
pSS: primary Sjögren’s syndrome
RA: rheumatoid arthritis
RORα: receptor-related orphan receptor α
RORγt: retinoid-related orphan receptor gamma T
SCFAs: short-chain fatty acids
scFv: single-chain variable fragment
SLE: systemic lupus erythematosus
SMAD: Sma and mad related proteins
STAT: signal transducer and activator of transcription
T1D: type 1 diabetes
Tconv: conventional T cells
TCR: T Cell Receptor
TCs: Thetis cells
Teff: T effector cells
Tfh: follicular T helper (Tfh) cells
Tfr: follicular regulatory T cells
TGF-β: transforming growth factor-beta
Th2: T helper 2 cell
TLR: toll-like receptor
TNF: tumor necrosis factor
TNFR: tumor necrosis factor receptor
Tr1: type 1 regulatory T cell
Tregs: regulatory T cells
TSLPR: thymic stromal lymphopoietin receptor
VIP: vasoactive intestinal peptide
WAS: Wiskott-Aldrich syndrome.

## References

[1] McInnes IB Gravallese EM . Immune-mediated inflammatory disease therapeutics: past, present and future. Nat Rev Immunol. (2021) 21:680–6. doi: 10.1038/s41577-021-00603-1, PMID: 34518662 PMC8436867

[2] Song Y Li J Wu Y . Evolving understanding of autoimmune mechanisms and new therapeutic strategies of autoimmune disorders. Signal Transduct Target Ther. (2024) 9:263. doi: 10.1038/s41392-024-01952-8, PMID: 39362875 PMC11452214

[3] Ogulur I Mitamura Y Yazici D Pat Y Ardicli S Li M . Type 2 immunity in allergic diseases. Cell Mol Immunol. (2025) 22:211–42. doi: 10.1038/s41423-025-01261-2, PMID: 39962262 PMC11868591

[4] Ramirez GA Cardamone C Lettieri S Fredi M Mormile I . Clinical and pathophysiological tangles between allergy and autoimmunity: deconstructing an old dichotomic paradigm. Clin Rev Allergy Immunol. (2025) 68:13. doi: 10.1007/s12016-024-09020-3, PMID: 39932658 PMC11814061

[5] Georgiev P Benamar M Han S Haigis MC Sharpe AH Chatila TA . Regulatory T cells in dominant immunologic tolerance. J Allergy Clin Immunol. (2024) 153:28–41. doi: 10.1016/j.jaci.2023.09.025, PMID: 37778472 PMC10842646

[6] Dikiy S Rudensky AY . Principles of regulatory T cell function. Immunity. (2023) 56:240–55. doi: 10.1016/j.immuni.2023.01.004, PMID: 36792571

[7] Lu Y Man XY . Diversity and function of regulatory T cells in health and autoimmune diseases. J Autoimmun. (2025) 151:103357. doi: 10.1016/j.jaut.2025.103357, PMID: 39805189

[8] Noval Rivas M Chatila TA . Regulatory T cells in allergic diseases. J Allergy Clin Immunol. (2016) 138:639–52. doi: 10.1016/j.jaci.2016.06.003, PMID: 27596705 PMC5023156

[9] Singer M Elsayed AM Husseiny MI . Regulatory T-cells: the face-off of the immune balance. Front Biosci (Landmark Ed). (2024) 29:377. doi: 10.31083/j.fbl2911377, PMID: 39614434

[10] Nedoszytko B Lange M Sokolowska-Wojdylo M Renke J Trzonkowski P Sobjanek M . The role of regulatory T cells and genes involved in their differentiation in pathogenesis of selected inflammatory and neoplastic skin diseases. Part I: Treg properties and functions. Postepy Dermatol Alergol. (2017) 34:285–94. doi: 10.5114/ada.2017.69305, PMID: 28951701 PMC5560174

[11] Bilate AM Lafaille JJ . Induced CD4^+^ Foxp3^+^ regulatory T cells in immune tolerance. Annu Rev Immunol. (2012) 30:733–58. doi: 10.1146/annurev-immunol-020711-075043, PMID: 22224762

[12] Lio C-WJ Hsieh C-S . Becoming self-aware: The thymic education of regulatory T cells. Curr Opin Immunol. (2011) 23:213–9. doi: 10.1016/j.coi.2010.11.010, PMID: 21146972 PMC3061250

[13] Chang L Wu H Huang W Li Y Chen Y Li X . IL-21 induces pyroptosis of treg cells via akt–mTOR–NLRP3–caspase 1 axis in eosinophilic chronic rhinosinusitis. J Allergy Clin Immunol. (2023) 152:641–655.e14. doi: 10.1016/j.jaci.2023.04.013, PMID: 37164271

[14] Shevach EM Thornton AM . tTregs, pTregs, and iTregs: similarities and differences. Immunol Rev. (2014) 259:88–102. doi: 10.1111/imr.12160, PMID: 24712461 PMC3982187

[15] Davidson TS DiPaolo RJ Andersson J Shevach EM . Cutting edge: IL-2 is essential for TGF-β-mediated induction of Foxp3+ T regulatory cells. J Immunol. (2007) 178:4022–6. doi: 10.4049/jimmunol.178.7.4022, PMID: 17371955

[16] Xiao S Jin H Korn T Liu SM Oukka M Lim B . Retinoic acid increases Foxp3+ regulatory T cells and inhibits development of Th17 cells by enhancing TGF-β-driven Smad3 signaling and inhibiting IL-6 and IL-23 receptor expression. J Immunol. (2008) 181:2277–84. doi: 10.4049/jimmunol.181.4.2277, PMID: 18684916 PMC2722959

[17] Hong J-Y Li S-S Hu T-Y Liu Z-Q Yu D Yu H-Q . Frontline science: TLR3 activation inhibits food allergy in mice by inducing IFN-γ+ Foxp3+ regulatory T cells. J Leukocyte Biol. (2019) 106:1201–9. doi: 10.1002/JLB.3HI0918-348RR, PMID: 30997942

[18] Xu LZ Xie RD Xie H Ju JY Fu XY Di DL . Chimeric specific antigen epitope-carrying dendritic cells induce interleukin-17(+) regulatory T cells to suppress food allergy. Clin Exp Allergy. (2020) 50:231–43. doi: 10.1111/cea.13528, PMID: 31715648

[19] Haribhai D Lin W Edwards B Ziegelbauer J Salzman NH Carlson MR . A central role for induced regulatory T cells in tolerance induction in experimental colitis. J Immunol. (2009) 182:3461–8. doi: 10.4049/jimmunol.0802535, PMID: 19265124 PMC2763205

[20] Kitani A Fuss I Nakamura K Kumaki F Usui T Strober W . Transforming growth factor (TGF)-β1–producing regulatory T cells induce smad-mediated interleukin 10 secretion that facilitates coordinated immunoregulatory activity and amelioration of TGF-β1–mediated fibrosis. J Exp Med. (2003) 198:1179–88. doi: 10.1084/jem.20030917, PMID: 14557415 PMC2194234

[21] Collison LW Chaturvedi V Henderson AL Giacomin PR Guy C Bankoti J . IL-35-mediated induction of a potent regulatory T cell population. Nat Immunol. (2010) 11:1093–101. doi: 10.1038/ni.1952, PMID: 20953201 PMC3008395

[22] Grossman WJ Verbsky JW Barchet W Colonna M Atkinson JP Ley TJ . Human T regulatory cells can use the perforin pathway to cause autologous target cell death. Immunity. (2004) 21:589–601. doi: 10.1016/j.immuni.2004.09.002, PMID: 15485635

[23] Gondek DC Lu L-F Quezada SA Sakaguchi S Noelle RJ . Cutting edge: Contact-mediated suppression by CD4+CD25+ regulatory cells involves a granzyme B-dependent, perforin-independent mechanism. J Immunol. (2005) 174:1783–6. doi: 10.4049/jimmunol.174.4.1783, PMID: 15699103

[24] Sula Karreci E Eskandari SK Dotiwala F Routray SK Kurdi AT Assaker JP . Human regulatory T cells undergo self-inflicted damage via granzyme pathways upon activation. JCI Insight. (2017) 2:e91599. doi: 10.1172/jci.insight.91599, PMID: 29093262 PMC5690280

[25] Thornton AM Shevach EM . CD4+CD25+ immunoregulatory T cells suppress polyclonal T cell activation *in vitro* by inhibiting interleukin 2 production. J Exp Med. (1998) 188:287–96. doi: 10.1084/jem.188.2.287, PMID: 9670041 PMC2212461

[26] Kim H-J Barnitz RA Kreslavsky T Brown FD Moffett H Lemieux ME . Stable inhibitory activity of regulatory T cells requires the transcription factor helios. Sci (New York N.Y.). (2015) 350:334–9. doi: 10.1126/science.aad0616, PMID: 26472910 PMC4627635

[27] Thornton AM Korty PE Tran DQ Wohlfert EA Murray PE Belkaid Y . Expression of Helios, an Ikaros transcription factor family member, differentiates thymic-derived from peripherally induced Foxp3+ T regulatory cells. J Immunol. (2010) 184:3433–41. doi: 10.4049/jimmunol.0904028, PMID: 20181882 PMC3725574

[28] Ruffo E Wu RC Bruno TC Workman CJ Vignali DAA . Lymphocyte-activation gene 3 (LAG3): The next immune checkpoint receptor. Semin Immunol. (2019) 42:101305. doi: 10.1016/j.smim.2019.101305, PMID: 31604537 PMC6920665

[29] Read S Malmström V Powrie F . Cytotoxic T lymphocyte–associated antigen 4 plays an essential role in the function of Cd25+Cd4+ regulatory cells that control intestinal inflammation. J Exp Med. (2000) 192:295–302. doi: 10.1084/jem.192.2.295, PMID: 10899916 PMC2193261

[30] Liang B Workman C Lee J Chew C Dale BM Colonna L . Regulatory T cells inhibit dendritic cells by lymphocyte activation gene-3 engagement of MHC class II. J Immunol. (2008) 180:5916–26. doi: 10.4049/jimmunol.180.9.5916, PMID: 18424711

[31] Deaglio S Dwyer KM Gao W Friedman D Usheva A Erat A . Adenosine generation catalyzed by CD39 and CD73 expressed on regulatory T cells mediates immune suppression. J Exp Med. (2007) 204:1257–65. doi: 10.1084/jem.20062512, PMID: 17502665 PMC2118603

[32] Wang ES Verano AL Nowak RP Yuan JC Donovan KA Eleuteri NA . Acute pharmacological degradation of Helios destabilizes regulatory T cells. Nat Chem Biol. (2021) 17:711–7. doi: 10.1038/s41589-021-00802-w, PMID: 34035522 PMC8162940

[33] Schiering C Krausgruber T Chomka A Fröhlich A Adelmann K Wohlfert EA . The alarmin IL-33 promotes regulatory T-cell function in the intestine. Nature. (2014) 513:564–8. doi: 10.1038/nature13577, PMID: 25043027 PMC4339042

[34] Bach JF . The effect of infections on susceptibility to autoimmune and allergic diseases. N Engl J Med. (2002) 347:911–20. doi: 10.1056/NEJMra020100, PMID: 12239261

[35] Eastaff-Leung N Mabarrack N Barbour A Cummins A Barry S . Foxp3+ regulatory T cells, Th17 effector cells, and cytokine environment in inflammatory bowel disease. J Clin Immunol. (2010) 30:80–9. doi: 10.1007/s10875-009-9345-1, PMID: 19936899

[36] Yao Y Chen C-L Yu D Liu Z . Roles of follicular helper and regulatory T cells in allergic diseases and allergen immunotherapy. Allergy. (2021) 76:456–70. doi: 10.1111/all.14639, PMID: 33098663

[37] Jutel M Akdis M Blaser K Akdis CA . Mechanisms of allergen specific immunotherapy – T-cell tolerance and more. Allergy. (2006) 61:796–807. doi: 10.1111/j.1398-9995.2006.01175.x, PMID: 16792576

[38] Roth‐Walter F Adcock IM Benito‐Villalvilla C Bianchini R Bjermer L Boyman O . Immune modulation via T regulatory cell enhancement: Disease-modifying therapies for autoimmunity and their potential for chronic allergic and inflammatory diseases—an EAACI position paper of the task force on immunopharmacology (TIPCO). Allergy. (2021) 76:90–113. doi: 10.1111/all.14478, PMID: 32593226

[39] Sage PT Sharpe AH . T follicular regulatory cells. Immunol Rev. (2016) 271:246–59. doi: 10.1111/imr.12411, PMID: 27088919

[40] Sage Peter T Tan Catherine L Freeman Gordon J Haigis M Sharpe Arlene H . Defective TFH cell function and increased TFR cells contribute to defective antibody production in aging. Cell Rep. (2015) 12:163–71. doi: 10.1016/j.celrep.2015.06.015, PMID: 26146074 PMC4504745

[41] Tsuji M Komatsu N Kawamoto S Suzuki K Kanagawa O Honjo T . Preferential generation of follicular B helper T cells from Foxp3+ T cells in gut peyer’s patches. Sci (New York N.Y.). (2009) 323:1488–92. doi: 10.1126/science.1169152, PMID: 19286559

[42] Koch MA Tucker-Heard GS Perdue NR Killebrew JR Urdahl KB Campbell DJ . The transcription factor T-bet controls regulatory T cell homeostasis and function during type 1 inflammation. Nat Immunol. (2009) 10:595–602. doi: 10.1038/ni.1731, PMID: 19412181 PMC2712126

[43] Chung Y Tanaka S Chu F Nurieva RI Martinez GJ Rawal S . Follicular regulatory T cells expressing Foxp3 and bcl-6 suppress germinal center reactions. Nat Med. (2011) 17:983–8. doi: 10.1038/nm.2426, PMID: 21785430 PMC3151340

[44] Trujillo-Ochoa JL Kazemian M Afzali B . The role of transcription factors in shaping regulatory T cell identity. Nat Rev Immunol. (2023) 23:842–56. doi: 10.1038/s41577-023-00893-7, PMID: 37336954 PMC10893967

[45] Dolsten GA Pritykin Y . Genomic analysis of Foxp3 function in regulatory T cells. J Immunol (Baltimore Md.: 1950). (2023) 210:880–7. doi: 10.4049/jimmunol.2200864, PMID: 36947819 PMC10037560

[46] Dominguez-Villar M Hafler DA . Regulatory T cells in autoimmune disease. Nat Immunol. (2018) 19:665–73. doi: 10.1038/s41590-018-0120-4, PMID: 29925983 PMC7882196

[47] McClymont SA Putnam AL Lee MR Esensten JH Liu W Hulme MA . Plasticity of human regulatory T cells in healthy subjects and patients with type 1 diabetes. J Immunol. (2011) 186:3918–26. doi: 10.4049/jimmunol.1003099, PMID: 21368230 PMC3091943

[48] Abdel-Gadir A Stephen-Victor E Gerber GK Noval Rivas M Wang S Harb H . Microbiota therapy acts via a regulatory T cell MyD88/RORγt pathway to suppress food allergy. Nat Med. (2019) 25:1164–74. doi: 10.1038/s41591-019-0461-z, PMID: 31235962 PMC6677395

[49] Turner JA Stephen-Victor E Wang S Rivas MN Abdel-Gadir A Harb H . Regulatory T cell-derived TGF-β1 controls multiple checkpoints governing allergy and autoimmunity. Immunity. (2020) 53:1331–2. doi: 10.1016/j.immuni.2020.11.011, PMID: 33326768 PMC7780880

[50] Tao B Ruan G Wang D Li Y Wang Z Yin G . Imbalance of Peripheral Th17 and Regulatory T Cells in Children with Allergic Rhinitis and Bronchial Asthma. Iran J Allergy Asthma Immunol. (2015) 14:273–9., PMID: 26546895

[51] Leonard C Montamat G Davril C Domingues O Hunewald O Revets D . Comprehensive mapping of immune tolerance yields a regulatory TNF receptor 2 signature in a murine model of successful fel d 1-specific immunotherapy using high-dose CpG adjuvant. Allergy. (2021) 76:2153–65. doi: 10.1111/all.14716, PMID: 33345329 PMC8359185

[52] Saxena V Lakhan R Iyyathurai J Bromberg JS . Mechanisms of exTreg induction. Eur J Immunol. (2021) 51:1956–67. doi: 10.1002/eji.202049123, PMID: 33975379 PMC8338747

[53] Voo KS Wang Y-H Santori FR Boggiano C Wang Y-H Arima K . Identification of IL-17-producing FOXP3+ regulatory T cells in humans. Proc Natl Acad Sci United States America. (2009) 106:4793–8. doi: 10.1073/pnas.0900408106, PMID: 19273860 PMC2653560

[54] Massoud AH Charbonnier L-M Lopez D Pellegrini M Phipatanakul W Chatila TA . An asthma-associated IL4R variant exacerbates airway inflammation by promoting conversion of regulatory T cells to TH17-like cells. Nat Med. (2016) 22:1013–22. doi: 10.1038/nm.4147, PMID: 27479084 PMC5014738

[55] Casaca VI Illi S Klucker E Ballenberger N Schedel M von Mutius E . STAT6 polymorphisms are associated with neonatal regulatory T cells and cytokines and atopic diseases at 3 years. Allergy. (2013) 68:1249–58. doi: 10.1111/all.12220, PMID: 24053457

[56] Malmhäll C Bossios A Pullerits T Lötvall J . Effects of pollen and nasal glucocorticoid on FOXP3^+^, GATA-3^+^ and T-bet^+^ cells in allergic rhinitis. Allergy. (2007) 62:1007–13. doi: 10.1111/j.1398-9995.2007.01420.x, PMID: 17686103

[57] Joudi AM Reyes Flores CP Singer BD . Epigenetic control of regulatory T cell stability and function: Implications for translation. Front Immunol. (2022) 13:861607. doi: 10.3389/fimmu.2022.861607, PMID: 35309306 PMC8924620

[58] Kawakami R Kitagawa Y Chen KY Arai M Ohara D Nakamura Y . Distinct Foxp3 enhancer elements coordinate development, maintenance, and function of regulatory T cells. Immunity. (2021) 54:947–961.e8. doi: 10.1016/j.immuni.2021.04.005, PMID: 33930308

[59] Wang L Liu Y Beier UH Han R Bhatti TR Akimova T . Foxp3+ T-regulatory cells require DNA methyltransferase 1 expression to prevent development of lethal autoimmunity. Blood. (2013) 121:3631–9. doi: 10.1182/blood-2012-08-451765, PMID: 23444399 PMC3643763

[60] Liu Y Wang L Han R Beier UH Akimova T Bhatti T . Two histone/protein acetyltransferases, CBP and p300, are indispensable for Foxp3^+^ T-regulatory cell development and function. Mol Cell Biol. (2014) 34:3993–4007. doi: 10.1128/MCB.00919-14, PMID: 25154413 PMC4386456

[61] Yang R Qu C Zhou Y Konkel JE Shi S Liu Y . Hydrogen sulfide promotes Tet1- and Tet2-mediated Foxp3 demethylation to drive regulatory T cell differentiation and maintain immune homeostasis. Immunity. (2015) 43:251–63. doi: 10.1016/j.immuni.2015.07.017, PMID: 26275994 PMC4731232

[62] Li Y Deuring J Peppelenbosch MP Kuipers EJ de Haar C van der Woude CJ . IL-6-induced DNMT1 activity mediates SOCS3 promoter hypermethylation in ulcerative colitis-related colorectal cancer. Carcinogenesis. (2012) 33:1889–96. doi: 10.1093/carcin/bgs214, PMID: 22739025

[63] Hori S Nomura T Sakaguchi S . Control of regulatory T cell development by the transcription factor Foxp3. Science. (2003) 299:1057–61. doi: 10.1126/science.1079490, PMID: 12522256

[64] He M Zong X Xu B Qi W Huang W Djekidel MN . Dynamic Foxp3-chromatin interaction controls tunable Treg cell function. J Exp Med. (2024) 221:e20232068. doi: 10.1084/jem.20232068, PMID: 38935023 PMC11211070

[65] Borna S Lee E Nideffer J Ramachandran A Wang B Baker J . Identification of unstable regulatory and autoreactive effector T cells that are expanded in patients with FOXP3 mutations. Sci Transl Med. (2023) 15:eadg6822. doi: 10.1126/scitranslmed.adg6822, PMID: 38117899 PMC11070150

[66] Goudy K Aydin D Barzaghi F Gambineri E Vignoli M Ciullini Mannurita S . Human IL2RA null mutation mediates immunodeficiency with lymphoproliferation and autoimmunity. Clin Immunol. (2013) 146:248–61. doi: 10.1016/j.clim.2013.01.004, PMID: 23416241 PMC3594590

[67] Kucuk ZY Charbonnier LM McMasters RL Chatila T Bleesing JJ . CTLA-4 haploinsufficiency in a patient with an autoimmune lymphoproliferative disorder. J Allergy Clin Immunol. (2017) 140:862–4. doi: 10.1016/j.jaci.2017.02.032, PMID: 28366794 PMC5591763

[68] Goswami TK Singh M Dhawan M Mitra S Emran TB Rabaan AA . Regulatory T cells (Tregs) and their therapeutic potential against autoimmune disorders - Advances and challenges. Hum Vaccin Immunother. (2022) 18:2035117. doi: 10.1080/21645515.2022.2035117, PMID: 35240914 PMC9009914

[69] Song X Chen R Li J Zhu Y Jiao J Liu H . Fragile Treg cells: Traitors in immune homeostasis? Pharmacol Res. (2024) 206:107297. doi: 10.1016/j.phrs.2024.107297, PMID: 38977207

[70] Rojas M Acosta-Ampudia Y Heuer LS Zang W D MM Ramirez-Santana C . Antigen-specific T cells and autoimmunity. J Autoimmun. (2024) 148:103303. doi: 10.1016/j.jaut.2024.103303, PMID: 39141985

[71] Patil S Gs V Sarode GS Sarode SC Khurayzi TA Mohamed Beshir SE . Exploring the role of immunotherapeutic drugs in autoimmune diseases: A comprehensive review. J Oral Biol Craniofac Res. (2021) 11:291–6. doi: 10.1016/j.jobcr.2021.02.009, PMID: 33948430 PMC8080637

[72] Prasad P Verma S Surbhi Ganguly NK Chaturvedi V Mittal SA . Rheumatoid arthritis: advances in treatment strategies. Mol Cell Biochem. (2023) 478:69–88. doi: 10.1007/s11010-022-04492-3, PMID: 35725992

[73] Baron KJ Turnquist HR . Clinical manufacturing of regulatory T cell products for adoptive cell therapy and strategies to improve therapeutic efficacy. Organogenesis. (2023) 19:2164159. doi: 10.1080/15476278.2022.2164159, PMID: 36681905 PMC9870008

[74] Wu D Luo Y Li T Zhao X Lv T Fang G . Systemic complications of rheumatoid arthritis: Focus on pathogenesis and treatment. Front Immunol. (2022) 13:1051082. doi: 10.3389/fimmu.2022.1051082, PMID: 36618407 PMC9817137

[75] Morita T Shima Y Wing JB Sakaguchi S Ogata A Kumanogoh A . The proportion of regulatory T cells in patients with rheumatoid arthritis: A meta-analysis. PloS One. (2016) 11:e0162306. doi: 10.1371/journal.pone.0162306, PMID: 27622457 PMC5021283

[76] Holzer MT Almanzar G Woidich R Hugle B Haas JP Prelog M . Mitigated suppressive function of regulatory T cells (Treg) upon Th17-inducing cytokines in oligo- and polyarticular Juvenile Idiopathic Arthritis (JIA) patients. Pediatr Rheumatol Online J. (2022) 20:26. doi: 10.1186/s12969-022-00680-z, PMID: 35410224 PMC8996624

[77] Jule AM Hoyt KJ Wei K Gutierrez-Arcelus M Taylor ML Ng J . Th1 polarization defines the synovial fluid T cell compartment in oligoarticular juvenile idiopathic arthritis. JCI Insight. (2021) 6:e149185. doi: 10.1172/jci.insight.149185, PMID: 34403374 PMC8492302

[78] Kotschenreuther K Yan S Kofler DM . Migration and homeostasis of regulatory T cells in rheumatoid arthritis. Front Immunol. (2022) 13:947636. doi: 10.3389/fimmu.2022.947636, PMID: 36016949 PMC9398455

[79] Komatsu N Okamoto K Sawa S Nakashima T Oh-hora M Kodama T . Pathogenic conversion of Foxp3+ T cells into TH17 cells in autoimmune arthritis. Nat Med. (2014) 20:62–8. doi: 10.1038/nm.3432, PMID: 24362934

[80] Yan S Kotschenreuther K Deng S Kofler DM . Regulatory T cells in rheumatoid arthritis: functions, development, regulation, and therapeutic potential. Cell Mol Life Sci. (2022) 79:533. doi: 10.1007/s00018-022-04563-0, PMID: 36173485 PMC9522664

[81] Zhang J Liu H Chen Y Liu H Zhang S Yin G . Augmenting regulatory T cells: new therapeutic strategy for rheumatoid arthritis. Front Immunol. (2024) 15:1312919. doi: 10.3389/fimmu.2024.1312919, PMID: 38322264 PMC10844451

[82] Dittrich-Salamon M Meyer A Yan S Steinbach-Knodgen E Kotschenreuther K Stahl D . Regulatory T cells from patients with rheumatoid arthritis are characterized by reduced expression of ikaros zinc finger transcription factors. Cells. (2022) 11:2171. doi: 10.3390/cells11142171, PMID: 35883614 PMC9316388

[83] Zafari P Yari K Mostafaei S Iranshahi N Assar S Fekri A . Analysis of Helios gene expression and Foxp3 TSDR methylation in the newly diagnosed Rheumatoid Arthritis patients. Immunol Invest. (2018) 47:632–42. doi: 10.1080/08820139.2018.1480029, PMID: 29851536

[84] Schnell JT Briviesca RL Kim T Charbonnier LM Henderson LA van Wijk F . The ‘T(reg) paradox’ in inflammatory arthritis. Nat Rev Rheumatol. (2025) 21:9–21. doi: 10.1038/s41584-024-01190-w, PMID: 39653758

[85] Ameer MA Chaudhry H Mushtaq J Khan OS Babar M Hashim T . An overview of systemic lupus erythematosus (SLE) pathogenesis, classification, and management. Cureus. (2022) 14:e30330. doi: 10.7759/cureus.30330, PMID: 36407159 PMC9662848

[86] Huang J Li X Zhu Q Wang M Xie Z Zhao T . Imbalance of Th17 cells, Treg cells and associated cytokines in patients with systemic lupus erythematosus: a meta-analysis. Front Immunol. (2024) 15:1425847. doi: 10.3389/fimmu.2024.1425847, PMID: 39086480 PMC11288813

[87] Zhang Y Yang W Li W Zhao Y . NLRP3 inflammasome: checkpoint connecting innate and adaptive immunity in autoimmune diseases. Front Immunol. (2021) 12:732933. doi: 10.3389/fimmu.2021.732933, PMID: 34707607 PMC8542789

[88] Zhao X Wang S Wang S Xie J Cui D . mTOR signaling: A pivotal player in Treg cell dysfunction in systemic lupus erythematosus. Clin Immunol. (2022) 245:109153. doi: 10.1016/j.clim.2022.109153, PMID: 36265758

[89] Zou H Ma S Li L Xia X Zhou Y Zhang R . Downregulation of circular RNA ETS1 promotes SLE activity and inhibits Treg cell differentiation through miR-1205/FoxP3 molecular axis. Int Immunopharmacol. (2024) 128:111539. doi: 10.1016/j.intimp.2024.111539, PMID: 38244519

[90] Blinova VG Vasilyev VI Rodionova EB Zhdanov DD . The role of regulatory T cells in the onset and progression of primary sjogren’s syndrome. Cells. (2023) 12:1359. doi: 10.3390/cells12101359, PMID: 37408193 PMC10216593

[91] Voskuhl RR MacKenzie-Graham A . Chronic experimental autoimmune encephalomyelitis is an excellent model to study neuroaxonal degeneration in multiple sclerosis. Front Mol Neurosci. (2022) 15:1024058. doi: 10.3389/fnmol.2022.1024058, PMID: 36340686 PMC9629273

[92] Hosseinalizadeh H Rabiee F Eghbalifard N Rajabi H Klionsky DJ Rezaee A . Regulating the regulatory T cells as cell therapies in autoimmunity and cancer. Front Med (Lausanne). (2023) 10:1244298. doi: 10.3389/fmed.2023.1244298, PMID: 37828948 PMC10565010

[93] Li R Li H Yang X Hu H Liu P Liu H . Crosstalk between dendritic cells and regulatory T cells: Protective effect and therapeutic potential in multiple sclerosis. Front Immunol. (2022) 13:970508. doi: 10.3389/fimmu.2022.970508, PMID: 36177043 PMC9513370

[94] Yang C Ma Y Lu Q Qu Y Li Y Cheng S . 2-Bromo-1,4-Naphthalenedione promotes CD8(+) T cell expansion and limits Th1/Th17 to mitigate experimental autoimmune encephalomyelitis. J Neuroinflamm. (2024) 21:181. doi: 10.1186/s12974-024-03172-x, PMID: 39068463 PMC11283727

[95] Cinier J Hubert M Besson L Di Roio A Rodriguez C Lombardi V . Recruitment and expansion of tregs cells in the tumor environment-how to target them? Cancers (Basel). (2021) 13:1850. doi: 10.3390/cancers13081850, PMID: 33924428 PMC8069615

[96] Akgul A Freguia CF Maddaloni M Hoffman C Voigt A Nguyen CQ . Treatment with a Lactococcus lactis that chromosomally express E. coli cfaI mitigates salivary flow loss in a Sjogren’s syndrome-like disease. Sci Rep. (2023) 13:19489. doi: 10.1038/s41598-023-46557-3, PMID: 37945636 PMC10636062

[97] Farrugia BL Melrose J . The glycosaminoglycan side chains and modular core proteins of heparan sulphate proteoglycans and the varied ways they provide tissue protection by regulating physiological processes and cellular behaviour. Int J Mol Sci. (2023) 24:14101. doi: 10.3390/ijms241814101, PMID: 37762403 PMC10531531

[98] Du Y Yang L Wang X Jiang N Zhou Y Chen R . Proteome profiling of experimental autoimmune encephalomyelitis mouse model and the effect of a SUMO E1 inhibitor. J Proteome Res. (2024) 23:5312–25. doi: 10.1021/acs.jproteome.4c00229, PMID: 39568369

[99] Baharlooi H Mansourabadi AH Minbashi Moeini M Mohamed Khosroshahi L Azimi M . Nucleic acids as novel therapeutic modalities to address multiple sclerosis onset and progression. Cell Mol Neurobiol. (2022) 42:2611–27. doi: 10.1007/s10571-021-01158-4, PMID: 34694513 PMC11421605

[100] Campanelli F Natale G Marino G Ghiglieri V Calabresi P . Striatal glutamatergic hyperactivity in Parkinson’s disease. Neurobiol Dis. (2022) 168:105697. doi: 10.1016/j.nbd.2022.105697, PMID: 35314319

[101] Liu Z Zhai XR Du ZS Xu FF Huang Y Wang XQ . Dopamine receptor D2 on CD4(+) T cells is protective against neuroinflammation and neurodegeneration in a mouse model of Parkinson’s disease. Brain Behav Immun. (2021) 98:110–21. doi: 10.1016/j.bbi.2021.08.220, PMID: 34403737

[102] Aniwan S Santiago P Loftus EV Jr Park SH . The epidemiology of inflammatory bowel disease in Asia and Asian immigrants to Western countries. United Eur Gastroenterol J. (2022) 10:1063–76. doi: 10.1002/ueg2.12350, PMID: 36479863 PMC9752270

[103] Negi S Saini S Tandel N Sahu K Mishra RPN Tyagi RK . Translating treg therapy for inflammatory bowel disease in humanized mice. Cells. (2021) 10:1847. doi: 10.3390/cells10081847, PMID: 34440615 PMC8393385

[104] Smilek DE Ehlers MR Nepom GT . Restoring the balance: immunotherapeutic combinations for autoimmune disease. Dis Model Mech. (2014) 7:503–13. doi: 10.1242/dmm.015099, PMID: 24795433 PMC4007402

[105] Shaikh H Vargas JG Mokhtari Z Jarick KJ Ulbrich M Mosca JP . Mesenteric lymph node transplantation in mice to study immune responses of the gastrointestinal tract. Front Immunol. (2021) 12:689896. doi: 10.3389/fimmu.2021.689896, PMID: 34381447 PMC8352558

[106] Ramos GP Papadakis KA . Mechanisms of disease: inflammatory bowel diseases. Mayo Clin Proc. (2019) 94:155–65. doi: 10.1016/j.mayocp.2018.09.013, PMID: 30611442 PMC6386158

[107] Lord JD Valliant-Saunders K Hahn H Thirlby RC Ziegler SF . Paradoxically increased FOXP3+ T cells in IBD do not preferentially express the isoform of FOXP3 lacking exon 2. Dig Dis Sci. (2012) 57:2846–55. doi: 10.1007/s10620-012-2292-3, PMID: 22736020 PMC3482978

[108] Braat H McGuirk P Ten Kate FJ Huibregtse I Dunne PJ Hommes DW . Prevention of experimental colitis by parenteral administration of a pathogen-derived immunomodulatory molecule. Gut. (2007) 56:351–7. doi: 10.1136/gut.2006.099861, PMID: 16952913 PMC1856816

[109] Peng X Rao G Li X Tong N Tian Y Fu X . Preclinical models for Type 1 Diabetes Mellitus - A practical approach for research. Int J Med Sci. (2023) 20:1644–61. doi: 10.7150/ijms.86566, PMID: 37859703 PMC10583179

[110] Riaz F Wei P Pan F . PPARs at the crossroads of T cell differentiation and type 1 diabetes. Front Immunol. (2023) 14:1292238. doi: 10.3389/fimmu.2023.1292238, PMID: 37928539 PMC10623333

[111] Zhou L He X Cai P Li T Peng R Dang J . Induced regulatory T cells suppress Tc1 cells through TGF-beta signaling to ameliorate STZ-induced type 1 diabetes mellitus. Cell Mol Immunol. (2021) 18:698–710. doi: 10.1038/s41423-020-00623-2, PMID: 33446887 PMC8027661

[112] Marek-Trzonkowska N Mysliwiec M Dobyszuk A Grabowska M Derkowska I Juscinska J . Therapy of type 1 diabetes with CD4(+)CD25(high)CD127-regulatory T cells prolongs survival of pancreatic islets - results of one year follow-up. Clin Immunol. (2014) 153:23–30. doi: 10.1016/j.clim.2014.03.016, PMID: 24704576

[113] Spanier JA Fung V Wardell CM Alkhatib MH Chen Y Swanson LA . Tregs with an MHC class II peptide-specific chimeric antigen receptor prevent autoimmune diabetes in mice. J Clin Invest. (2023) 133:e168601. doi: 10.1172/JCI168601, PMID: 37561596 PMC10503798

[114] Yang SJ Singh AK Drow T Tappen T Honaker Y Barahmand-Pour-Whitman F . Pancreatic islet-specific engineered T(regs) exhibit robust antigen-specific and bystander immune suppression in type 1 diabetes models. Sci Transl Med. (2022) 14:eabn1716. doi: 10.1126/scitranslmed.abn1716, PMID: 36197963

[115] Kakabadse D Chen D Fishman S Weinstein-Marom H Davies J Wen L . Regulatory CD4(+) T cells redirected against pathogenic CD8(+) T cells protect NOD mice from development of autoimmune diabetes. Front Immunol. (2024) 15:1463971. doi: 10.3389/fimmu.2024.1463971, PMID: 39351219 PMC11439686

[116] Huang Q Zhu J . Regulatory T cell-based therapy in type 1 diabetes: Latest breakthroughs and evidence. Int Immunopharmacol. (2024) 140:112724. doi: 10.1016/j.intimp.2024.112724, PMID: 39098233

[117] Jmii H Fisson S Aouni M Jaidane H . Type B coxsackieviruses and central nervous system disorders: critical review of reported associations. Rev Med Virol. (2021) 31:e2191. doi: 10.1002/rmv.2191, PMID: 33159700

[118] Anders HJ Fernandez-Juarez GM Vaglio A Romagnani P Floege J . CKD therapy to improve outcomes of immune-mediated glomerular diseases. Nephrol Dial Transplant. (2023) 38:ii50–7. doi: 10.1093/ndt/gfad069, PMID: 37218706

[119] Li F Liang Z Zhong H Hu X Tang Z Zhu C . Group 3 innate lymphoid cells exacerbate lupus nephritis by promoting B cell activation in kidney ectopic lymphoid structures. Adv Sci (Weinh). (2023) 10:e2302804. doi: 10.1002/advs.202302804, PMID: 37915129 PMC10724443

[120] Linke A Tiegs G Neumann K . Pathogenic T-cell responses in immune-mediated glomerulonephritis. Cells. (2022) 11:1625. doi: 10.3390/cells11101625, PMID: 35626662 PMC9139939

[121] Wang YM Zhang GY Wang Y Hu M Wu H Watson D . Foxp3-transduced polyclonal regulatory T cells protect against chronic renal injury from adriamycin. J Am Soc Nephrol. (2006) 17:697–706. doi: 10.1681/ASN.2005090978, PMID: 16467443

[122] Liu J Gu QH Cui Z Zhao MH Jia XY . Short-chain fatty acids ameliorate experimental anti-glomerular basement membrane disease. Clin Immunol. (2024) 259:109903. doi: 10.1016/j.clim.2024.109903, PMID: 38218211

[123] Fouka E Drakopanagiotakis F Steiropoulos P . Pathogenesis of pulmonary manifestations in ANCA-associated vasculitis and goodpasture syndrome. Int J Mol Sci. (2024) 25:5278. doi: 10.3390/ijms25105278, PMID: 38791316 PMC11121030

[124] Corridon PR . Still finding ways to augment the existing management of acute and chronic kidney diseases with targeted gene and cell therapies: Opportunities and hurdles. Front Med (Lausanne). (2023) 10:1143028. doi: 10.3389/fmed.2023.1143028, PMID: 36960337 PMC10028138

[125] Zhang Z Zhu T Zhang L Xing Y Yan Z Li Q . Critical influence of cytokines and immune cells in autoimmune gastritis. Autoimmunity. (2023) 56:2174531. doi: 10.1080/08916934.2023.2174531, PMID: 36762543

[126] Khalilollah S Kalantari Soltanieh S Obaid Saleh R Ali Alzahrani A Ghaleb Maabreh H Mazin Al-Hamdani M . LncRNAs involvement in pathogenesis of immune-related disease via regulation of T regulatory cells, an updated review. Cytokine. (2024) 179:156585. doi: 10.1016/j.cyto.2024.156585, PMID: 38579428

[127] Qin Y Qian Y Liu S Chen R . A double-edged sword role of IFN-gamma-producing iNKT cells in sepsis: Persistent suppression of Treg cell formation in an Nr4a1-dependent manner. iScience. (2024) 27:111462. doi: 10.1016/j.isci.2024.111462, PMID: 39720538 PMC11667017

[128] Ni R Fan L Zhang L Song Y Wang H Wang A . A mouse model of irradiation and spleen-thymus lymphocyte infusion induced aplastic anemia. Hematology. (2022) 27:932–45. doi: 10.1080/16078454.2022.2113356, PMID: 36004514

[129] Chen Y Tong X Lu R Zhang Z Ma T . All-trans retinoic acid in hematologic disorders: not just acute promyelocytic leukemia. Front Pharmacol. (2024) 15:1404092. doi: 10.3389/fphar.2024.1404092, PMID: 39027338 PMC11254857

[130] Wang Y Li J Nakahata S Iha H . Complex role of regulatory T cells (Tregs) in the tumor microenvironment: their molecular mechanisms and bidirectional effects on cancer progression. Int J Mol Sci. (2024) 25:7346. doi: 10.3390/ijms25137346, PMID: 39000453 PMC11242872

[131] Xu YD Cheng M Shang PP Yang YQ . Role of IL-6 in dendritic cell functions. J Leukoc Biol. (2022) 111:695–709. doi: 10.1002/JLB.3MR0621-616RR, PMID: 34405445

[132] Zhong Y Lu TT Liu XM Liu BL Hu Y Liu S . High levels of thyroid hormone impair regulatory T cell function via reduced PD-1 expression. J Clin Endocrinol Metab. (2021) 106:2738–53. doi: 10.1210/clinem/dgab191, PMID: 33758937

[133] Raugh A Allard D Bettini M . Nature vs. nurture: FOXP3, genetics, and tissue environment shape Treg function. Front Immunol. (2022) 13:911151. doi: 10.3389/fimmu.2022.911151, PMID: 36032083 PMC9411801

[134] Knoedler S Knoedler L Kauke-Navarro M Rinkevich Y Hundeshagen G Harhaus L . Regulatory T cells in skin regeneration and wound healing. Mil Med Res. (2023) 10:49. doi: 10.1186/s40779-023-00484-6, PMID: 37867188 PMC10591349

[135] Pietraforte I Frasca L . Autoreactive T-cells in psoriasis: are they spoiled tregs and can therapies restore their functions? Int J Mol Sci. (2023) 24:4348. doi: 10.3390/ijms24054348, PMID: 36901778 PMC10002349

[136] Nussbaum L Chen YL Ogg GS . Role of regulatory T cells in psoriasis pathogenesis and treatment. Br J Dermatol. (2021) 184:14–24. doi: 10.1111/bjd.19380, PMID: 32628773

[137] Siroux V Boudier A Nadif R Lupinek C Valenta R Bousquet J . Association between asthma, rhinitis, and conjunctivitis multimorbidities with molecular IgE sensitization in adults. Allergy. (2019) 74:824–7. doi: 10.1111/all.13676, PMID: 30474280

[138] Melone G Giorgis V Di Pino M Pelaia C Nappi E Heffler E . Local allergic rhinitis: Lights and shadows of a mysterious entity. Int Arch Allergy Immunol. (2023) 184:12–20. doi: 10.1159/000526604, PMID: 36223735

[139] Akdis CA Arkwright PD Brüggen M-C Busse W Gadina M Guttman-Yassky E . Type 2 immunity in the skin and lungs. Allergy. (2020) 75:1582–605. doi: 10.1111/all.14318, PMID: 32319104

[140] Zhang Y Lan F Zhang L . Update on pathomechanisms and treatments in allergic rhinitis. Allergy. (2022) 77:3309–19. doi: 10.1111/all.15454, PMID: 35892225

[141] Palomares O Akdis M Martín-Fontecha M Akdis CA . Mechanisms of immune regulation in allergic diseases: The role of regulatory T and B cells. Immunol Rev. (2017) 278:219–36. doi: 10.1111/imr.12555, PMID: 28658547

[142] Huang F Yin J-N Wang H-B Liu S-Y Li Y-N . Association of imbalance of effector T cells and regulatory cells with the severity of asthma and allergic rhinitis in children. Allergy Asthma Proc. (2017) 38:70–7. doi: 10.2500/aap.2017.38.4076, PMID: 29046188

[143] Huang X Chen Y Zhang F Yang Q Zhang G . Peripheral Th17/treg cell-mediated immunity imbalance in allergic rhinitis patients. Braz J Otorhinolaryngology. (2014) 80:152–5. doi: 10.5935/1808-8694.20140031, PMID: 24830974 PMC9443953

[144] Genc S Eroglu H Kucuksezer UC Aktas-Cetin E Gelincik A Ustyol-Aycan E . The decreased CD4+CD25+ FoxP3+ T cells in nonstimulated allergic rhinitis patients sensitized to house dust mites. J Asthma: Off J Assoc Care Asthma. (2012) 49:569–74. doi: 10.3109/02770903.2012.695418, PMID: 22793523

[145] Liu W Ouyang H Zeng Q Luo R Lu G . Decreased treg-derived miR-181a and miR-155 correlated with reduced number and function of treg cells in allergic rhinitis children. Eur Arch oto-rhino-laryngology: Off J Eur Fed Oto-Rhino-Laryngological Societies (EUFOS): affiliated German Soc Oto-Rhino-Laryngology - Head Neck Surg. (2019) 276:1089–94. doi: 10.1007/s00405-019-05304-z, PMID: 30673848

[146] Ling EM Smith T Nguyen XD Pridgeon C Dallman M Arbery J . Relation of CD4+CD25+ regulatory T-cell suppression of allergen-driven T-cell activation to atopic status and expression of allergic disease. Lancet (London England). (2004) 363:608–15. doi: 10.1016/S0140-6736(04)15592-X, PMID: 14987885

[147] Jiao W-E Sun L Xu S Deng Y-Q Qiao Y-L Xi Y . NotcH2 suppresses the development of allergic rhinitis by promoting FOXP3 expression and treg cell differentiation. Life Sci. (2021) 284:119922. doi: 10.1016/j.lfs.2021.119922, PMID: 34480930

[148] Sun R Tang X-Y Yang Y . Immune imbalance of regulatory T/type 2 helper cells in the pathogenesis of allergic rhinitis in children. J Laryngology Otology. (2016) 130:89–94. doi: 10.1017/S0022215115003096, PMID: 26620633

[149] Sogut A Yilmaz O Kirmaz C Ozbilgin K Onur E Celik O . Regulatory-T, T-helper 1, and T-helper 2 cell differentiation in nasal mucosa of allergic rhinitis with olive pollen sensitivity. Int Arch Allergy Immunol. (2012) 157:349–53. doi: 10.1159/000329159, PMID: 22123238

[150] Marques CR Costa RS Costa GNDO da Silva TM Teixeira TO de Andrade EMM . Genetic and epigenetic studies of FOXP3 in asthma and allergy. Asthma Res Pract. (2015) 1:10. doi: 10.1186/s40733-015-0012-4, PMID: 27965764 PMC5142332

[151] Boonpiyathad T Sokolowska M Morita H Rückert B Kast JI Wawrzyniak M . Der p 1-specific regulatory T-cell response during house dust mite allergen immunotherapy. Allergy. (2019) 74:976–85. doi: 10.1111/all.13684, PMID: 30485456

[152] Ulges A Klein M Reuter S Gerlitzki B Hoffmann M Grebe N . Protein kinase CK2 enables regulatory T cells to suppress excessive TH2 responses *in vivo*. Nat Immunol. (2015) 16:267–75. doi: 10.1038/ni.3083, PMID: 25599562

[153] Wang YX Gu ZW Cao ZW . Difference between CD25(+)Tregs and Helios(+)Tregs in a murine model of allergic rhinitis. Braz J Otorhinolaryngol. (2021) 87:550–6. doi: 10.1016/j.bjorl.2019.12.001, PMID: 31974056 PMC9422529

[154] Shamji MH Layhadi JA Achkova D Kouser L Perera-Webb A Couto-Francisco NC . Role of IL-35 in sublingual allergen immunotherapy. J Allergy Clin Immunol. (2019) 143:1131–1142.e4. doi: 10.1016/j.jaci.2018.06.041, PMID: 30053528

[155] Cloots RHE Poynter ME Terwindt E Lamers WH Kohler SE . Hypoargininemia exacerbates airway hyperresponsiveness in a mouse model of asthma. Respir Res. (2018) 19:98. doi: 10.1186/s12931-018-0809-9, PMID: 29792217 PMC5967058

[156] Sokol K Sur S Ameredes BT . Inhaled environmental allergens and toxicants as determinants of the asthma phenotype. Adv Exp Med Biol. (2014) 795:43–73. doi: 10.1007/978-1-4614-8603-9_4, PMID: 24162902 PMC9116436

[157] Son SE Koh JM Im DS . Activation of free fatty acid receptor 4 (FFA4) ameliorates ovalbumin-induced allergic asthma by suppressing activation of dendritic and mast cells in mice. Int J Mol Sci. (2022) 23:5270. doi: 10.3390/ijms23095270, PMID: 35563671 PMC9100770

[158] Komlosi ZI van de Veen W Kovacs N Szucs G Sokolowska M O'Mahony L . Cellular and molecular mechanisms of allergic asthma. Mol Aspects Med. (2022) 85:100995. doi: 10.1016/j.mam.2021.100995, PMID: 34364680

[159] Suto A Nakajima H Kagami SI Suzuki K Saito Y Iwamoto I . Role of CD4(+) CD25(+) regulatory T cells in T helper 2 cell-mediated allergic inflammation in the airways. Am J Respir Crit Care Med. (2001) 164:680–7. doi: 10.1164/ajrccm.164.4.2010170, PMID: 11520737

[160] Tsai Y-G Yang KD Wen Y-S Hung C-H Chien J-W Lin C-Y . Allergen-specific immunotherapy enhances CD8+ CD25+ CD137+ regulatory T cells and decreases nasal nitric oxide. Pediatr Allergy Immunology: Off Publ Eur Soc Pediatr Allergy Immunol. (2019) 30:531–9. doi: 10.1111/pai.13061, PMID: 30968455

[161] Yao Y Wang Z-C Wang N Zhou P-C Chen C-L Song J . Allergen immunotherapy improves defective follicular regulatory T cells in patients with allergic rhinitis. J Allergy Clin Immunol. (2019) 144:118–28. doi: 10.1016/j.jaci.2019.02.008, PMID: 30796979

[162] Cheng X Lou W Wang C Zhang W Han D Zhang L . FOXP3-marked IL-17a-producing regulatory T cells are increased in patients with allergic rhinitis. Acta Oto-Laryngologica. (2012) 132:1311–7. doi: 10.3109/00016489.2012.709320, PMID: 22992221

[163] Lippitsch A Baal N Chukovetskyi Y Cunningham S Michel G Dietert K . Plasmacytoid dendritic cell depletion modifies FoxP3+ T cell homeostasis and the clinical course of bacterial pneumonia in mice. J Leukocyte Biol. (2019) 106:977–85. doi: 10.1002/JLB.3AB0119-014RR, PMID: 31265764

[164] Soroosh P Doherty TA Duan W Mehta AK Choi H Adams YF . Lung-resident tissue macrophages generate Foxp3+ regulatory T cells and promote airway tolerance. J Exp Med. (2013) 210:775–88. doi: 10.1084/jem.20121849, PMID: 23547101 PMC3620360

[165] Hartl D Koller B Mehlhorn AT Reinhardt D Nicolai T Schendel DJ . Quantitative and functional impairment of pulmonary CD4+CD25hi regulatory T cells in pediatric asthma. J Allergy Clin Immunol. (2007) 119:1258–66. doi: 10.1016/j.jaci.2007.02.023, PMID: 17412402

[166] Nguyen KD Vanichsarn C Fohner A Nadeau KC . Selective deregulation in chemokine signaling pathways of CD4+CD25(hi)CD127(lo)/(-) regulatory T cells in human allergic asthma. J Allergy Clin Immunol. (2009) 123:933–939.e10. doi: 10.1016/j.jaci.2008.11.037, PMID: 19152963 PMC4214553

[167] Chantveerawong T Sangkangjanavanich S Chiewchalermsri C Pradubpongsa P Mitthamsiri W Jindarat S . Increased circulating CRTH2+ tregs are associated with asthma control and exacerbation. Allergy. (2022) 77:681–5. doi: 10.1111/all.15145, PMID: 34676900

[168] Kraszula Ł Eusebio M-O Kuna P Pietruczuk M . Relationship between CCR5+FoxP3+ treg cells and forced expiratory volume in 1 s, peak expiratory flow in patients with severe asthma. Postepy Dermatologii I Alergologii. (2021) 38:262–8. doi: 10.5114/ada.2021.106202, PMID: 34408594 PMC8362744

[169] Hew KM Walker AI Kohli A Garcia M Syed A McDonald-Hyman C . Childhood exposure to ambient polycyclic aromatic hydrocarbons is linked to epigenetic modifications and impaired systemic immunity in T cells. Clin Exp Allergy. (2015) 45:238–48. doi: 10.1111/cea.12377, PMID: 25048800 PMC4396982

[170] Prunicki M Stell L Dinakarpandian D de Planell-Saguer M Lucas RW Hammond SK . Exposure to NO(2), CO, and PM(2.5) is linked to regional DNA methylation differences in asthma. Clin Epigenet. (2018) 10:2. doi: 10.1186/s13148-017-0433-4, PMID: 29317916 PMC5756438

[171] Nadeau K McDonald-Hyman C Noth EM Pratt B Hammond SK Balmes J . Ambient air pollution impairs regulatory T-cell function in asthma. J Allergy Clin Immunol. (2010) 126:845–852.e10. doi: 10.1016/j.jaci.2010.08.008, PMID: 20920773

[172] Schaub B Liu J Höppler S Schleich I Huehn J Olek S . Maternal farm exposure modulates neonatal immune mechanisms through regulatory T cells. J Allergy Clin Immunol. (2009) 123:774–782.e5. doi: 10.1016/j.jaci.2009.01.056, PMID: 19348917

[173] Benamar M Harb H Chen Q Wang M Chan TMF Fong J . A common IL-4 receptor variant promotes asthma severity via a treg cell GRB2-IL-6-Notch4 circuit. Allergy. (2022) 77:3377–87. doi: 10.1111/all.15444, PMID: 35841382 PMC9617759

[174] Doganci A Eigenbrod T Krug N De Sanctis GT Hausding M Erpenbeck VJ . The IL-6R alpha chain controls lung CD4+CD25+ treg development and function during allergic airway inflammation *in vivo*. J Clin Invest. (2005) 115:313–25. doi: 10.1172/JCI200522433, PMID: 15668741 PMC544603

[175] Esty B Harb H Bartnikas LM Charbonnier LM Massoud AH Leon-Astudillo C . Treatment of severe persistent asthma with IL-6 receptor blockade. J Allergy Clin Immunology: In Pract. (2019) 7:1639–1642.e4. doi: 10.1016/j.jaip.2019.02.043, PMID: 30885880 PMC6511285

[176] MacBeth M Joetham A Gelfand EW Schedel M . Plasticity of naturally occurring regulatory T cells in allergic airway disease is modulated by the transcriptional activity of il-6. Int J Mol Sci. (2021) 22:4582. doi: 10.3390/ijms22094582, PMID: 33925531 PMC8123826

[177] Branum AM Lukacs SL . Food allergy among children in the United States. Pediatrics. (2009) 124:1549–55. doi: 10.1542/peds.2009-1210, PMID: 19917585

[178] Baseggio Conrado A Ierodiakonou D Gowland MH Boyle RJ Turner PJ . Food anaphylaxis in the United Kingdom: Analysis of national data, 1998-2018. BMJ. (2021) 372:n251. doi: 10.1136/bmj.n251, PMID: 33597169 PMC7885259

[179] Feng H Xiong X Chen Z Xu Q Zhang Z Luo N . Prevalence and influencing factors of food allergy in global context: A meta-analysis. Int Arch Allergy Immunol. (2023) 184:320–52. doi: 10.1159/000527870, PMID: 36634638

[180] Warren CM Jiang J Gupta RS . Epidemiology and burden of food allergy. Curr Allergy Asthma Rep. (2020) 20:6. doi: 10.1007/s11882-020-0898-7, PMID: 32067114 PMC7883751

[181] Spolidoro GCI Amera YT Ali MM Nyassi S Lisik D Ioannidou A . Frequency of food allergy in europe: An updated systematic review and meta-analysis. Allergy. (2023) 78:351–68. doi: 10.1111/all.15560, PMID: 36271775 PMC10099188

[182] Vitaliti G Cimino C Coco A Pratico AD Lionetti E . The immunopathogenesis of cow’s milk protein allergy (CMPA). Ital J Pediatr. (2012) 38:35. doi: 10.1186/1824-7288-38-35, PMID: 22824011 PMC3441837

[183] Wu S Zhang R Liu Y Gao J Wu Y Tu C . *In vitro* effect of flavonoids on basophils degranulation and intestinal epithelial barrier damage induced by omega-5 gliadin-derived peptide. Foods. (2022) 11:3857. doi: 10.3390/foods11233857, PMID: 36496664 PMC9741160

[184] Spergel JM . From atopic dermatitis to asthma: the atopic march. Ann Allergy Asthma Immunol. (2010) 105:99–106. doi: 10.1016/j.anai.2009.10.002, PMID: 20674819

[185] Kanagaratham C El Ansari YS Lewis OL Oettgen HC . IgE and IgG antibodies as regulators of mast cell and basophil functions in food allergy. Front Immunol. (2020) 11:603050. doi: 10.3389/fimmu.2020.603050, PMID: 33362785 PMC7759531

[186] Sampson HA O'Mahony L Burks AW Plaut M Lack G Akdis CA . Mechanisms of food allergy. J Allergy Clin Immunol. (2018) 141:11–9. doi: 10.1016/j.jaci.2017.11.005, PMID: 29307410

[187] Palomares O . The role of regulatory T cells in IgE-mediated food allergy. J Investigational Allergology Clin Immunol. (2013) 23:371–82., PMID: 24459813

[188] Sicherer SH Warren CM Dant C Gupta RS Nadeau KC . Food allergy from infancy through adulthood. J Allergy Clin Immunology: In Pract. (2020) 8:1854–64. doi: 10.1016/j.jaip.2020.02.010, PMID: 32499034 PMC7899184

[189] Chinthrajah RS Hernandez JD Boyd SD Galli SJ Nadeau KC . Molecular and cellular mechanisms of food allergy and food tolerance. J Allergy Clin Immunol. (2016) 137:984–97. doi: 10.1016/j.jaci.2016.02.004, PMID: 27059726 PMC5030841

[190] Noval Rivas M Burton Oliver T Wise P Charbonnier L-M Georgiev P Oettgen Hans C . Regulatory T cell reprogramming toward a TH2-cell-like lineage impairs oral tolerance and promotes food allergy. Immunity. (2015) 42:512–23. doi: 10.1016/j.immuni.2015.02.004, PMID: 25769611 PMC4366316

[191] Shin HS Shon DH . Food and natural materials target mechanisms to effectively regulate allergic responses. J Nutr Sci Vitaminol (Tokyo). (2015) 61 Suppl:S109–11. doi: 10.3177/jnsv.61.S109, PMID: 26598817

[192] Yamashita H Takahashi K Tanaka H Nagai H Inagaki N . Overcoming food allergy through acquired tolerance conferred by transfer of tregs in a murine model. Allergy. (2012) 67:201–9. doi: 10.1111/j.1398-9995.2011.02742.x, PMID: 22050332

[193] Prince BT Devonshire AL Erickson KA Bergerson J Fuleihan D Szychlinski C . Regulatory T-cell populations in children are affected by age and food allergy diagnosis. J Allergy Clin Immunol. (2017) 140:1194–1196.e16. doi: 10.1016/j.jaci.2017.04.039, PMID: 28549988 PMC5777506

[194] Chinthrajah RS Purington N Sampath V Andorf S Manohar M Prunicki M . High dimensional immune biomarkers demonstrate differences in phenotypes and endotypes in food allergy and asthma. Ann Allergy Asthma Immunol. (2018) 121:117–119.e1. doi: 10.1016/j.anai.2018.04.022, PMID: 29705381 PMC6026562

[195] Shreffler WG Wanich N Moloney M Nowak-Wegrzyn A Sampson HA . Association of allergen-specific regulatory T cells with the onset of clinical tolerance to milk protein. J Allergy Clin Immunol. (2009) 123:43–52.e7. doi: 10.1016/j.jaci.2008.09.051, PMID: 19130927

[196] Syed A Garcia MA Lyu S-C Bucayu R Kohli A Ishida S . Peanut oral immunotherapy results in increased antigen-induced regulatory T-cell function and hypomethylation of forkhead box protein 3 (FOXP3). J Allergy Clin Immunol. (2014) 133:500–510.e11. doi: 10.1016/j.jaci.2013.12.1037, PMID: 24636474 PMC4121175

[197] Wang M Yang IV Davidson EJ Joetham A Takeda K O'Connor BP . Forkhead box protein 3 demethylation is associated with tolerance induction in peanut-induced intestinal allergy. J Allergy Clin Immunol. (2018) 141:659–670.e2. doi: 10.1016/j.jaci.2017.04.020, PMID: 28479331 PMC5671381

[198] Akagbosu B Tayyebi Z Shibu G Paucar Iza YA Deep D Parisotto YF . Novel antigen-presenting cell imparts treg-dependent tolerance to gut microbiota. Nature. (2022) 610:752–60. doi: 10.1038/s41586-022-05309-5, PMID: 36070798 PMC9605865

[199] Kedmi R Najar TA Mesa KR Grayson A Kroehling L Hao Y . A RORγt+ cell instructs gut microbiota-specific treg cell differentiation. Nature. (2022) 610:737–43. doi: 10.1038/s41586-022-05089-y, PMID: 36071167 PMC9908423

[200] Lyu M Suzuki H Kang L Gaspal F Zhou W Goc J . ILC3s select microbiota-specific regulatory T cells to establish tolerance in the gut. Nature. (2022) 610:744–51. doi: 10.1038/s41586-022-05141-x, PMID: 36071169 PMC9613541

[201] Verma R Lee C Jeun E-J Yi J Kim KS Ghosh A . Cell surface polysaccharides of bifidobacterium bifidum induce the generation of Foxp3^+^ regulatory T cells. Sci Immunol. (2018) 3:eaat6975. doi: 10.1126/sciimmunol.aat6975, PMID: 30341145

[202] Song X Sun X Oh SF Wu M Zhang Y Zheng W . Microbial bile acid metabolites modulate gut RORγ+ regulatory T cell homeostasis. Nature. (2020) 577:410–5. doi: 10.1038/s41586-019-1865-0, PMID: 31875848 PMC7274525

[203] Silverberg JI Hanifin JM . Adult eczema prevalence and associations with asthma and other health and demographic factors: A US population-based study. J Allergy Clin Immunol. (2013) 132:1132–8. doi: 10.1016/j.jaci.2013.08.031, PMID: 24094544

[204] Newell L Polak ME Perera J Owen C Boyd P Pickard C . Sensitization via healthy skin programs TH2 responses in individuals with atopic dermatitis. J Invest Dermatol. (2013) 133:2372–80. doi: 10.1038/jid.2013.148, PMID: 23528819

[205] Werfel T Allam J-P Biedermann T Eyerich K Gilles S Guttman-Yassky E . Cellular and molecular immunologic mechanisms in patients with atopic dermatitis. J Allergy Clin Immunol. (2016) 138:336–49. doi: 10.1016/j.jaci.2016.06.010, PMID: 27497276

[206] Tan BB Weald D Strickland I Friedmann PS . Double-blind controlled trial of effect of housedust-mite allergen avoidance on atopic dermatitis. Lancet (London England). (1996) 347:15–8. doi: 10.1016/S0140-6736(96)91556-1, PMID: 8531541

[207] Schuler CF Tsoi LC Billi AC Harms PW Weidinger S Gudjonsson JE . Genetic and immunological pathogenesis of atopic dermatitis. J Invest Dermatol. (2024) 144:954–68. doi: 10.1016/j.jid.2023.10.019, PMID: 38085213 PMC11040454

[208] Sroka-Tomaszewska J Trzeciak M . Molecular mechanisms of atopic dermatitis pathogenesis. Int J Mol Sci. (2021) 22:4130. doi: 10.3390/ijms22084130, PMID: 33923629 PMC8074061

[209] Tsakok T Woolf R Smith CH Weidinger S Flohr C . Atopic dermatitis: The skin barrier and beyond. Br J Dermatol. (2019) 180:464–74. doi: 10.1111/bjd.16934, PMID: 29969827

[210] Scharschmidt TC Vasquez KS Truong H-A Gearty SV Pauli ML Nosbaum A . A wave of regulatory T cells into neonatal skin mediates tolerance to commensal microbes. Immunity. (2015) 43:1011–21. doi: 10.1016/j.immuni.2015.10.016, PMID: 26588783 PMC4654993

[211] Scharschmidt TC Vasquez KS Pauli ML Leitner EG Chu K Truong H-A . Commensal microbes and hair follicle morphogenesis coordinately drive treg migration into neonatal skin. Cell Host Microbe. (2017) 21:467–477.e5. doi: 10.1016/j.chom.2017.03.001, PMID: 28343820 PMC5516645

[212] Harrison OJ Linehan JL Shih H-Y Bouladoux N Han S-J Smelkinson M . Commensal-specific T cell plasticity promotes rapid tissue adaptation to injury. Science. (2019) 363:eaat6280. doi: 10.1126/science.aat6280, PMID: 30523076 PMC7304459

[213] Kalekar LA Cohen JN Prevel N Sandoval PM Mathur AN Moreau JM . Regulatory T cells in skin are uniquely poised to suppress profibrotic immune responses. Sci Immunol. (2019) 4:eaaw2910. doi: 10.1126/sciimmunol.aaw2910, PMID: 31492709 PMC6848056

[214] Malhotra N Leyva-Castillo JM Jadhav U Barreiro O Kam C O'Neill NK . RORα-expressing T regulatory cells restrain allergic skin inflammation. Sci Immunol. (2018) 3:eaao6923. doi: 10.1126/sciimmunol.aao6923, PMID: 29500225 PMC5912895

[215] Kashiwagi M Hosoi J Lai J-F Brissette J Ziegler SF Morgan BA . Direct control of regulatory T cells by keratinocytes. Nat Immunol. (2017) 18:334–43. doi: 10.1038/ni.3661, PMID: 28092372 PMC5310986

[216] Delacher M Imbusch CD Weichenhan D Breiling A Hotz-Wagenblatt A Träger U . Genome-wide DNA-methylation landscape defines specialization of regulatory T cells in tissues. Nat Immunol. (2017) 18:1160–72. doi: 10.1038/ni.3799, PMID: 28783152 PMC5912503

[217] Agrawal R Wisniewski JA Woodfolk JA . The role of regulatory T cells in atopic dermatitis. Curr Probl Dermatol. (2011) 41:112–24. doi: 10.1159/000323305, PMID: 21576952 PMC4547455

[218] Bacchetta R Barzaghi F Roncarolo MG . From IPEX syndrome to FOXP3 mutation: a lesson on immune dysregulation. Ann N Y Acad Sci. (2018) 1417:5–22. doi: 10.1111/nyas.13011, PMID: 26918796

[219] Nahm D-H . Regulatory T cell-targeted immunomodulatory therapy for long-term clinical improvement of atopic dermatitis: Hypotheses and perspectives. Life (Basel Switzerland). (2023) 13:1674. doi: 10.3390/life13081674, PMID: 37629531 PMC10455293

[220] Ochs HD Thrasher AJ . The wiskott-aldrich syndrome. J Allergy Clin Immunol. (2006) 117:725–38. doi: 10.1016/j.jaci.2006.02.005, PMID: 16630926

[221] Fisher SA Rahimzadeh M Brierley C Gration B Doree C Kimber CE . The role of vitamin D in increasing circulating T regulatory cell numbers and modulating T regulatory cell phenotypes in patients with inflammatory disease or in healthy volunteers: A systematic review. PloS One. (2019) 14:e0222313. doi: 10.1371/journal.pone.0222313, PMID: 31550254 PMC6759203

[222] Li Q Zhou Q Zhang G Tian X Li Y Wang Z . Vitamin D supplementation and allergic diseases during childhood: A systematic review and meta-analysis. Nutrients. (2022) 14:3947. doi: 10.3390/nu14193947, PMID: 36235600 PMC9571357

[223] Ali N Zirak B Rodriguez RS Pauli ML Truong H-A Lai K . Regulatory T cells in skin facilitate epithelial stem cell differentiation. Cell. (2017) 169:1119–1129.e11. doi: 10.1016/j.cell.2017.05.002, PMID: 28552347 PMC5504703

[224] Sanchez Rodriguez R Pauli ML Neuhaus IM Yu SS Arron ST Harris HW . Memory regulatory T cells reside in human skin. J Clin Invest. (2014) 124:1027–36. doi: 10.1172/JCI72932, PMID: 24509084 PMC3934172

[225] Ogonowska P Gilaberte Y Barańska-Rybak W Nakonieczna J . Colonization with staphylococcus aureus in atopic dermatitis patients: Attempts to reveal the unknown. Front Microbiol. (2021) 11:567090. doi: 10.3389/fmicb.2020.567090, PMID: 33505363 PMC7830525

[226] Byrd AL Deming C Cassidy SKB Harrison OJ Ng W-I Conlan S . Staphylococcus aureus and staphylococcus epidermidis strain diversity underlying pediatric atopic dermatitis. Sci Trans Med. (2017) 9:eaal4651. doi: 10.1126/scitranslmed.aal4651, PMID: 28679656 PMC5706545

[227] Kim KJ Ha J Kim SW Kim JE Lee S Choi HS . Bone loss after solid organ transplantation: A review of organ-specific considerations. Endocrinol Metab (Seoul). (2024) 39:267–82. doi: 10.3803/EnM.2024.1939, PMID: 38693817 PMC11066446

[228] Sanchez-Leon ML Jimenez-Cortegana C Cabrera G Vermeulen EM dela Cruz-Merino L Sanchez-Margalet V . The effects of dendritic cell-based vaccines in the tumor microenvironment: Impact on myeloid-derived suppressor cells. Front Immunol. (2022) 13:1050484. doi: 10.3389/fimmu.2022.1050484, PMID: 36458011 PMC9706090

[229] Taves MD Ashwell JD . Glucocorticoids in T cell development, differentiation and function. Nat Rev Immunol. (2021) 21:233–43. doi: 10.1038/s41577-020-00464-0, PMID: 33149283

[230] Collins CP Khuat LT Sckisel GD Vick LV Minnar CM Dunai C . Systemic immunostimulation induces glucocorticoid-mediated thymic involution succeeded by rebound hyperplasia which is impaired in aged recipients. Front Immunol. (2024) 15:1429912. doi: 10.3389/fimmu.2024.1429912, PMID: 39315105 PMC11416920

[231] Garcia-Lacarte M Grijalba SC Melchor J Arnaiz-Leche A Roa S . The PD-1/PD-L1 checkpoint in normal germinal centers and diffuse large B-cell lymphomas. Cancers (Basel). (2021) 13:4683. doi: 10.3390/cancers13184683, PMID: 34572910 PMC8471895

[232] Liang Y Wei J Shen J Liang Z Ma X Du Y . Immunological pathogenesis and treatment progress of adenovirus pneumonia in children. Ital J Pediatr. (2025) 51:4. doi: 10.1186/s13052-024-01836-1, PMID: 39789604 PMC11715079

[233] Ding M Jin L Zhao J Yang L Cui S Wang X . Add-on sirolimus for the treatment of mild or moderate systemic lupus erythematosus via T lymphocyte subsets balance. Lupus Sci Med. (2024) 11:e001072. doi: 10.1136/lupus-2023-001072, PMID: 38351097 PMC10868177

[234] Cheung J Zahorowska B Suranyi M Wong JKW Diep J Spicer ST . CD4(+)CD25(+) T regulatory cells in renal transplantation. Front Immunol. (2022) 13:1017683. doi: 10.3389/fimmu.2022.1017683, PMID: 36426347 PMC9681496

[235] Karagiannidis C Akdis M Holopainen P Woolley NJ Hense G Ruckert B . Glucocorticoids upregulate FOXP3 expression and regulatory T cells in asthma. J Allergy Clin Immunol. (2004) 114:1425–33. doi: 10.1016/j.jaci.2004.07.014, PMID: 15577848

[236] Zuska-Prot M Maslanka T . Effect of inhaled and systemic glucocorticoid treatment on CD4(+) regulatory and effector T cells in a mouse model of allergic asthma. Int Immunopharmacol. (2017) 45:98–109. doi: 10.1016/j.intimp.2017.02.005, PMID: 28189974

[237] Cari L De Rosa F Nocentini G Riccardi C . Context-dependent effect of glucocorticoids on the proliferation, differentiation, and apoptosis of regulatory T cells: A review of the empirical evidence and clinical applications. Int J Mol Sci. (2019) 20:1142. doi: 10.3390/ijms20051142, PMID: 30845709 PMC6429178

[238] Wang N Zhou K Liang Z Sun R Tang H Yang Z . RapaLink-1 outperforms rapamycin in alleviating allogeneic graft rejection by inhibiting the mTORC1-4E-BP1 pathway in mice. Int Immunopharmacol. (2023) 125:111172. doi: 10.1016/j.intimp.2023.111172, PMID: 37951193

[239] Rivera A Heitman J . Natural product ligands of FKBP12: Immunosuppressive antifungal agents FK506, rapamycin, and beyond. PloS Pathog. (2023) 19:e1011056. doi: 10.1371/journal.ppat.1011056, PMID: 36634035 PMC9836287

[240] Cassano A Chong AS Alegre ML . Tregs in transplantation tolerance: role and therapeutic potential. Front Transplant. (2023) 2:1217065. doi: 10.3389/frtra.2023.1217065, PMID: 38993904 PMC11235334

[241] Bulliard Y Freeborn R Uyeda MJ Humes D Bjordahl R de Vries D . From promise to practice: CAR T and Treg cell therapies in autoimmunity and other immune-mediated diseases. Front Immunol. (2024) 15:1509956. doi: 10.3389/fimmu.2024.1509956, PMID: 39697333 PMC11653210

[242] Hammond S Thomson P Meng X Naisbitt D . *In-vitro* approaches to predict and study T-cell mediated hypersensitivity to drugs. Front Immunol. (2021) 12:630530. doi: 10.3389/fimmu.2021.630530, PMID: 33927714 PMC8076677

[243] Zhong HL Li PZ Li D Guan CX Zhou Y . The role of vasoactive intestinal peptide in pulmonary diseases. Life Sci. (2023) 332:122121. doi: 10.1016/j.lfs.2023.122121, PMID: 37742737

[244] Montgomery S Miedema MD Dodson JA . Aspirin and statin therapy for primary prevention of cardiovascular disease in older adults. Heart. (2022) 108:1090–7. doi: 10.1136/heartjnl-2021-320154, PMID: 34764212 PMC11977457

[245] Greca E Kacimi O Poudel S Wireko AA Abdul-Rahman T Michel G . Immunomodulatory effect of different statin regimens on regulatory T-cells in patients with acute coronary syndrome: a systematic review and network meta-analysis of randomized clinical trials. Eur Heart J Cardiovasc Pharmacother. (2023) 9:122–8. doi: 10.1093/ehjcvp/pvac047, PMID: 36047962

[246] Al-Qahtani AA Alhamlan FS Al-Qahtani AA . Pro-inflammatory and anti-inflammatory interleukins in infectious diseases: A comprehensive review. Trop Med Infect Dis. (2024) 9:13. doi: 10.3390/tropicalmed9010013, PMID: 38251210 PMC10818686

[247] Harris F Berdugo YA Tree T . IL-2-based approaches to Treg enhancement. Clin Exp Immunol. (2023) 211:149–63. doi: 10.1093/cei/uxac105, PMID: 36399073 PMC10019135

[248] Lokau J Petasch LM Garbers C . The soluble IL-2 receptor alpha/CD25 as a modulator of IL-2 function. Immunology. (2024) 171:377–87. doi: 10.1111/imm.13723, PMID: 38037265

[249] Permanyer M Bosnjak B Glage S Friedrichsen M Floess S Huehn J . Efficient IL-2R signaling differentially affects the stability, function, and composition of the regulatory T-cell pool. Cell Mol Immunol. (2021) 18:398–414. doi: 10.1038/s41423-020-00599-z, PMID: 33408345 PMC8027001

[250] Hu X Li J Fu M Zhao X Wang W . The JAK/STAT signaling pathway: from bench to clinic. Signal Transduct Target Ther. (2021) 6:402. doi: 10.1038/s41392-021-00791-1, PMID: 34824210 PMC8617206

[251] Overwijk WW Tagliaferri MA Zalevsky J . Engineering IL-2 to give new life to T cell immunotherapy. Annu Rev Med. (2021) 72:281–311. doi: 10.1146/annurev-med-073118-011031, PMID: 33158368

[252] Ko B Takebe N Andrews O Makena MR Chen AP . Rethinking oncologic treatment strategies with interleukin-2. Cells. (2023) 12:1316. doi: 10.3390/cells12091316, PMID: 37174716 PMC10177415

[253] Rosenzwajg M Lorenzon R Cacoub P Pham HP Pitoiset F El Soufi K . Immunological and clinical effects of low-dose interleukin-2 across 11 autoimmune diseases in a single, open clinical trial. Ann Rheumatic Dis. (2019) 78:209–17. doi: 10.1136/annrheumdis-2018-214229, PMID: 30472651

[254] Zhang X Miao M Zhang R Liu X Zhao X Shao M . Efficacy and safety of low-dose interleukin-2 in combination with methotrexate in patients with active rheumatoid arthritis: a randomized, double-blind, placebo-controlled phase 2 trial. Signal Transduct Target Ther. (2022) 7:67. doi: 10.1038/s41392-022-00887-2, PMID: 35250032 PMC8898945

[255] Kohler C Smole U Kratzer B Trapin D Schmetterer KG Pickl WF . Allergen alters IL-2/alphaIL-2-based Treg expansion but not tolerance induction in an allergen-specific mouse model. Allergy. (2020) 75:1618–29. doi: 10.1111/all.14203, PMID: 31991489 PMC7383865

[256] Bonnet B Vigneron J Levacher B Vazquez T Pitoiset F Brimaud F . Low-dose IL-2 induces regulatory T cell-mediated control of experimental food allergy. J Immunol. (2016) 197:188–98. doi: 10.4049/jimmunol.1501271, PMID: 27259854

[257] Rosenzwajg M Salet R Lorenzon R Tchitchek N Roux A Bernard C . Low-dose IL-2 in children with recently diagnosed type 1 diabetes: a Phase I/II randomised, double-blind, placebo-controlled, dose-finding study. Diabetologia. (2020) 63:1808–21. doi: 10.1007/s00125-020-05200-w, PMID: 32607749

[258] Medler J Kucka K Wajant H . Tumor necrosis factor receptor 2 (TNFR2): an emerging target in cancer therapy. Cancers (Basel). (2022) 14:2603. doi: 10.3390/cancers14112603, PMID: 35681583 PMC9179537

[259] Mensink M Verleng LJ Schrama E Janssen GM Tjokrodirijo RT van Veelen PA . Tregs from human blood differentiate into nonlymphoid tissue-resident effector cells upon TNFR2 costimulation. JCI Insight. (2024) 9:e172942. doi: 10.1172/jci.insight.172942, PMID: 38341270 PMC10972588

[260] Bai L Hao X Keith J Feng Y . DNA methylation in regulatory T cell differentiation and function: challenges and opportunities. Biomolecules. (2022) 12:1282. doi: 10.3390/biom12091282, PMID: 36139121 PMC9496199

[261] Urbano PCM He X van Heeswijk B Filho OPS Tijssen H Smeets RL . TNFalpha-signaling modulates the kinase activity of human effector treg and regulates IL-17A expression. Front Immunol. (2019) 10:3047. doi: 10.3389/fimmu.2019.03047, PMID: 32038615 PMC6986271

[262] Chen K Gu X Yang S Tao R Fan M Bao W . Research progress on intestinal tissue-resident memory T cells in inflammatory bowel disease. Scand J Immunol. (2023) 98:e13332. doi: 10.1111/sji.13332, PMID: 38441381

[263] Brinkman N McCann K Gooch B . The purification of plasma proteins for therapeutic use. In: Rossi’s principles of transfusion medicine, 6th edition. Hoboken: John Wiley & Sons, Inc. (2022). p. 216–35.

[264] Jandus C Jandus P . Effects of intravenous immunoglobulins on human innate immune cells: collegium internationale allergologicum update 2024. Int Arch Allergy Immunol. (2024) 185:975–96. doi: 10.1159/000539069, PMID: 38852585

[265] Bayry J Ahmed EA Toscano-Rivero D Vonniessen N Genest G Cohen CG . Intravenous immunoglobulin: mechanism of action in autoimmune and inflammatory conditions. J Allergy Clin Immunol Pract. (2023) 11:1688–97. doi: 10.1016/j.jaip.2023.04.002, PMID: 37062358

[266] Javidan M Amiri AM Koohi N Joudaki N Bashirrohelleh MA Pirsadeghi A . Restoring immune balance with Tregitopes: A new approach to treating immunological disorders. BioMed Pharmacother. (2024) 177:116983. doi: 10.1016/j.biopha.2024.116983, PMID: 38908205

[267] Kedzierska AE Lorek D Slawek A Chelmonska-Soyta A . Tregitopes regulate the tolerogenic immune response and decrease the foetal death rate in abortion-prone mouse matings. Sci Rep. (2020) 10:10531. doi: 10.1038/s41598-020-66957-z, PMID: 32601347 PMC7324366

[268] De Groot AS Khan S Mattei AE Lelias S Martin WD . Does human homology reduce the potential immunogenicity of non-antibody scaffolds? Front Immunol. (2023) 14:1215939. doi: 10.3389/fimmu.2023.1215939, PMID: 38022550 PMC10664710

[269] Ishina IA Zakharova MY Kurbatskaia IN Mamedov AE Belogurov AA Jr Gabibov AG . MHC class II presentation in autoimmunity. Cells. (2023) 12:314. doi: 10.3390/cells12020314, PMID: 36672249 PMC9856717

[270] Bemani P Jalili S Hassanpour K Faraji F Gholijani N Barazesh M . Designing and characterization of tregitope-based multi-epitope vaccine against multiple sclerosis: an immunoinformatic approach. Curr Drug Saf. (2023) 18:79–92. doi: 10.2174/1574886317666220429105439, PMID: 35507799

[271] Tian X Zhao J Song Y Wang Q Li M Liu J . 2022 Chinese guideline for the management of pregnancy and reproduction in systemic lupus erythematosus. Rheumatol Immunol Res. (2023) 4:115–38. doi: 10.2478/rir-2023-0019, PMID: 37781682 PMC10538620

[272] Pandey SP Bhaskar R Han SS Narayanan KB . Autoimmune responses and therapeutic interventions for systemic lupus erythematosus: A comprehensive review. Endocr Metab Immune Disord Drug Targets. (2024) 24:499–518. doi: 10.2174/1871530323666230915112642, PMID: 37718519

[273] Dembele M Tao S Massoud AH Miah SMS Lelias S De Groot AS . Tregitopes improve asthma by promoting highly suppressive and antigen-specific tregs. Front Immunol. (2021) 12:634509. doi: 10.3389/fimmu.2021.634509, PMID: 33953711 PMC8089381

[274] Honing DY Luiten RM Matos TR . Regulatory T cell dysfunction in autoimmune diseases. Int J Mol Sci. (2024) 25:7171. doi: 10.3390/ijms25137171, PMID: 39000278 PMC11241405

[275] Bittner S Hehlgans T Feuerer M . Engineered Treg cells as putative therapeutics against inflammatory diseases and beyond. Trends Immunol. (2023) 44:468–83. doi: 10.1016/j.it.2023.04.005, PMID: 37100644

[276] Li YL Hung WC . Reprogramming of sentinel lymph node microenvironment during tumor metastasis. J BioMed Sci. (2022) 29:84. doi: 10.1186/s12929-022-00868-1, PMID: 36266717 PMC9583492

[277] Carballido JM Regairaz C Rauld C Raad L Picard D Kammuller M . The emerging jamboree of transformative therapies for autoimmune diseases. Front Immunol. (2020) 11:472. doi: 10.3389/fimmu.2020.00472, PMID: 32296421 PMC7137386

[278] Baeten P Van Zeebroeck L Kleinewietfeld M Hellings N Broux B . Improving the efficacy of regulatory T cell therapy. Clin Rev Allergy Immunol. (2022) 62:363–81. doi: 10.1007/s12016-021-08866-1, PMID: 34224053 PMC8256646

[279] Romano M Fanelli G Albany CJ Giganti G Lombardi G . Past, present, and future of regulatory T cell therapy in transplantation and autoimmunity. Front Immunol. (2019) 10:43. doi: 10.3389/fimmu.2019.00043, PMID: 30804926 PMC6371029

[280] Christofi P Pantazi C Psatha N Sakellari I Yannaki E Papadopoulou A . Promises and pitfalls of next-generation treg adoptive immunotherapy. Cancers (Basel). (2023) 15:5877. doi: 10.3390/cancers15245877, PMID: 38136421 PMC10742252

[281] Janssens I Cools N . Regulating the regulators: Is introduction of an antigen-specific approach in regulatory T cells the next step to treat autoimmunity? Cell Immunol. (2020) 358:104236. doi: 10.1016/j.cellimm.2020.104236, PMID: 33137651

[282] Uenishi GI Repic M Yam JY Landuyt A Saikumar-Lakshmi P Guo T . GNTI-122: an autologous antigen-specific engineered Treg cell therapy for type 1 diabetes. JCI Insight. (2024) 9:e171844. doi: 10.1172/jci.insight.171844, PMID: 38516892 PMC11063937

[283] Martin-Cruz L Benito-Villalvilla C Sirvent S Angelina A Palomares O . The role of regulatory T cells in allergic diseases: collegium internationale allergologicum (CIA) update 2024. Int Arch Allergy Immunol. (2024) 185:503–18. doi: 10.1159/000536335, PMID: 38408438

[284] Tuomela K Levings MK . Genetic engineering of regulatory T cells for treatment of autoimmune disorders including type 1 diabetes. Diabetologia. (2024) 67:611–22. doi: 10.1007/s00125-023-06076-2, PMID: 38236408

[285] Gozalvez E Lario A Munoz-Sanchez G Lozano F . Regulatory T cell-based adoptive cell therapy in autoimmunity. Int J Mol Sci. (2025) 26:10340. doi: 10.3390/ijms262110340, PMID: 41226379 PMC12610882

[286] Kohlgruber AC Dezfulian MH Sie BM Wang CI Kula T Laserson U . High-throughput discovery of MHC class I- and II-restricted T cell epitopes using synthetic cellular circuits. Nat Biotechnol. (2025) 43:623–34. doi: 10.1038/s41587-024-02248-6, PMID: 38956325 PMC11994455

[287] Sakaguchi S Kawakami R Mikami N . Treg-based immunotherapy for antigen-specific immune suppression and stable tolerance induction: a perspective. Immunother Adv. (2023) 3:ltad007. doi: 10.1093/immadv/ltad007, PMID: 37397971 PMC10309084

[288] Amini L Greig J Schmueck-Henneresse M Volk HD Bezie S Reinke P . Super-treg: toward a new era of adoptive treg therapy enabled by genetic modifications. Front Immunol. (2020) 11:611638. doi: 10.3389/fimmu.2020.611638, PMID: 33717052 PMC7945682

[289] Tay C Tanaka A Sakaguchi S . Tumor-infiltrating regulatory T cells as targets of cancer immunotherapy. Cancer Cell. (2023) 41:450–65. doi: 10.1016/j.ccell.2023.02.014, PMID: 36917950

[290] Sadeqi Nezhad M Seifalian A Bagheri N Yaghoubi S Karimi MH Adbollahpour-Alitappeh M . Chimeric antigen receptor based therapy as a potential approach in autoimmune diseases: how close are we to the treatment? Front Immunol. (2020) 11:603237. doi: 10.3389/fimmu.2020.603237, PMID: 33324420 PMC7727445

[291] Sarmay G . Biologia Futura: Emerging antigen-specific therapies for autoimmune diseases. Biol Futur. (2021) 72:15–24. doi: 10.1007/s42977-021-00074-4, PMID: 34554499

[292] Barden M Holzinger A Velas L Mezosi-Csaplar M Szoor A Vereb G . CAR and TCR form individual signaling synapses and do not cross-activate, however, can co-operate in T cell activation. Front Immunol. (2023) 14:1110482. doi: 10.3389/fimmu.2023.1110482, PMID: 36817444 PMC9929185

[293] Abdeladhim M Zhang AH Kropp LE Lindrose AR Venkatesha SH Mitre E . Engineered ovalbumin-expressing regulatory T cells protect against anaphylaxis in ovalbumin-sensitized mice. Clin Immunol. (2019) 207:49–54. doi: 10.1016/j.clim.2019.07.009, PMID: 31325629 PMC6742773

[294] Schmetterer KG Haiderer D Leb-Reichl VM Neunkirchner A Jahn-Schmid B Kung HJ . Bet v 1-specific T-cell receptor/forkhead box protein 3 transgenic T cells suppress Bet v 1-specific T-cell effector function in an activation-dependent manner. J Allergy Clin Immunol. (2011) 127:238–45. doi: 10.1016/j.jaci.2010.10.023, PMID: 21211658

[295] Depil S Duchateau P Grupp SA Mufti G Poirot L . ‘Off-the-shelf’ allogeneic CAR T cells: development and challenges. Nat Rev Drug Discov. (2020) 19:185–99. doi: 10.1038/s41573-019-0051-2, PMID: 31900462

[296] Caldwell KJ Gottschalk S Talleur AC . Allogeneic CAR cell therapy-more than a pipe dream. Front Immunol. (2020) 11:618427. doi: 10.3389/fimmu.2020.618427, PMID: 33488631 PMC7821739

